# Microcalorimetric Investigations of Reversible Staphylococcal Enterotoxin Unfolding

**DOI:** 10.3390/toxins14080554

**Published:** 2022-08-15

**Authors:** Susan C. Berry, Odbert A. Triplett, Li-Rong Yu, Mark E. Hart, Lauren S. Jackson, William H. Tolleson

**Affiliations:** 1 National Center for Toxicological Research, US Food and Drug Administration, 3900 NCTR Road, Jefferson, AR 72079, USA; 2 Division of Food Processing Science & Technology, Center for Food Safety and Applied Nutrition, US Food and Drug Administration, 6502 S. Archer Rd., Bedford Park, IL 60501, USA

**Keywords:** differential scanning calorimetry, Staphylococcal enterotoxins, protein unfolding, food safety

## Abstract

Staphylococcal food poisoning (SFP) is a common food-borne illness often associated with contamination during food handling. The genes for Staphylococcal enterotoxin (SE) isoforms SEA and SEB are frequently detected in human nasal *Staphylococcus aureus* isolates and these toxins are commonly associated with SFP. Past studies described the resistance of preformed SE proteins to heat inactivation and their reactivation upon cooling in foods. Full thermodynamic analyses for these processes have not been reported, however. The thermal stabilities of SEA, SEB, and SEH and reversibility of unfolding in simple buffers were investigated at pH 4.5 and pH 6.8 using differential scanning calorimetry (DSC). SEA and SEB unfolding was irreversible at pH 6.8 and at least partially reversible at pH 4.5 while SEH unfolding was irreversible at pH 4.5 and reversible at pH 6.8. Additional studies showed maximum refolding for SEB at pH 3.5–4.0 and diminished refolding at pH 4.5 with increasing ionic strength. SE-stimulated secretion of interferon-gamma by human peripheral blood mononuclear cells was used to assess residual SE biological activity following heat treatments using conditions matching those used for DSC studies. The biological activities of SEB and SEH exhibited greater resistance to heat inactivation than that of SEA. The residual activities of heat-treated SEB and SEH were measurable but diminished further in the presence of reconstituted nonfat dry milk adjusted to pH 4.5 or pH 6.8. To different extents, the pH and ionic strengths typical for foods influenced the thermal stabilities of SEA, SEB, and SEH and their potentials to renature spontaneously after heat treatments.

## 1. Introduction

Staphylococcal food poisoning (SFP) associated with strains of *Staphylococcus aureus* and related species producing one or more members of the Staphylococcal enterotoxin (SE) protein family is one of the most common food-borne illnesses worldwide [1]. The Staphylococcal enterotoxins are a family of 25 protein exotoxins that possess two biological activities associated with their potent toxicity—emesis and superantigenicity [2,3,4]. The former accounts for the violent gastrointestinal syndrome associated with acute SFP outbreaks and the latter is responsible for the immunotoxicity associated with SE exposure. SE superantigenicity is due to its ability to act as a scaffold to engage the β-chain variable (Vβ) region of the T-cell receptor (TCR) of T-lymphocytes while simultaneously binding the major histocompatibility complex (MHC-II) proteins of antigen-presenting cells independently of the specific antigens normally required for that interaction. Although all SE proteins can engage both MHC-II and Vβ TCR extracellular domains, individual SE isoforms exhibit different binding specificities for these targets [5]. In this way, MHC-II/Vβ TCR crosslinking by SE binding in the absence of specific antigens stimulates a greater population of lymphocytes to release large amounts of interferon-γ (IFNG) and other inflammatory cytokines to produce a toxic shock response that can be fatal in some cases. The SE protein family consists of five well studied classical isoforms, SEA-SEE, which are considered Category B Select Agents of bioterrorism by the US Department of Health and Human Services, along with an additional 20 novel SE or Staphylococcal enterotoxin-like isoforms (SEG-SElX) that are not designated as Select Agents [1]. Importantly, SFP has been associated with *S. aureus* isolates that express one or more of the novel SE isoforms in the absence of the five classical isoforms [6,7,8].

Most SFP cases appear to be linked to the contamination of foods during handling [9]. Foods most often implicated in SFP include meat products, poultry and egg products, milk and dairy products, salads, cream-filled pastries, and sandwich fillings [10]. Certain types of foods present additional risks for SFP. For example, bovine mastitis in dairy cattle may lead to the contamination of raw milk by *S. aureus* [1,11] and SFP outbreaks have been linked to dairy products in the past [12,13,14]. This is also complicated by the fact that milk is a good substrate for *S. aureus* growth and enterotoxin production [11]. Surprisingly low levels of SE contamination are associated with food poisoning, with a toxic dose estimated to be 20–100 ng SE/individual [1]. An SFP outbreak in Japan was linked to reconstituted low fat skim milk contaminated with ≤0.38 ng/mL SEA [12]. An earlier SFP outbreak in the US was associated with chocolate milk containing 0.40–0.78 ng/mL SEA [15].

In addition to their extreme biological potencies, the potential for SE-contaminated foods to cause SFP is exacerbated by their remarkable thermal stabilities. Raw milk is typically pasteurized prior to consumption in fluid milk products and before processing into other foods and ingredients such as cheese, ice cream, and powdered milk. Appropriate time and temperature conditions for thermal processing established for foods, such as raw milk, are designed to reduce or eliminate hazardous biological agents. Resistance to heat inactivation is a notable characteristic associated with SE proteins. Their ability to persist through heat treatments that can eliminate Staphylococci allows preformed toxins to induce SFP even in the absence of viable bacteria. Moreover, the reactivation of heat-treated SEA, SEB, and SEC in foods has been reported [16,17]. The thermal stabilities for most novel SE isoforms and their potential for reactivation after heat treatments have not been investigated as thoroughly as they have for the classical SE isoforms. Laboratory-based studies are needed to identify the processing conditions as well as physical and chemical properties of food that influence inactivation and reactivation of these novel SE isoforms.

Differential scanning calorimetry (DSC) is a biophysical instrumental technique uniquely suited to study thermal stabilities of proteins and evaluate the reversibility of their unfolding. Microcalorimeters designed for this application measure differences in heat capacities between sample and reference cells with high precision while scanning temperature ranges typical for reversible transitions associated with biological macromolecules, such as protein unfolding (0–100 °C). Key thermodynamic parameters determined from DSC thermograms include the observed (or calorimetric) enthalpy (ΔH_cal_), calorimetric entropy (ΔS_cal_), and melting temperature (T_m_) associated with the transition. Fundamental thermodynamic relationships also allow Gibbs energies (ΔG) and equilibrium constants (K_eq_) to be derived from DSC thermograms. Theoretical van’t Hoff enthalpies (ΔH_vH_) can be determined from the temperature dependence of K_eq_ observed by DSC as it scans through the temperature range involved in the protein unfolding process. In addition, comparisons of ΔH_cal_ to ΔH_vH_ provide further information regarding potential folding intermediates or cooperative melting [18,19]. Using this approach, DSC has been applied in studies of the *Corynebacterium* diphtheria toxin T-domain [20], *S. aureus* exfoliative toxin D-like protein [21], *Escherichia coli* Shiga toxin B subunit [22], *Abrus precatorius* abrin II [23], and *Ricinus communis* ricin [24]. However, DSC has been applied only once in the study of SE toxins. Yanaka et al. [25] utilized DSC to investigate the role of a flexible region present in typical and mutant forms of SEB on protein stability. Only enthalpies and melting temperatures were reported in this study, meaning that a thorough thermodynamic analysis for SE unfolding using DSC has not yet been reported for any isoform. DSC was applied in the current study to compare the thermal stabilities of SEA, SEB, and recombinant SEH and the reversibility of thermal unfolding under various conditions.

## 2. Results

### 2.1. Staphylococcal Enterotoxin H

**Figure 1:**
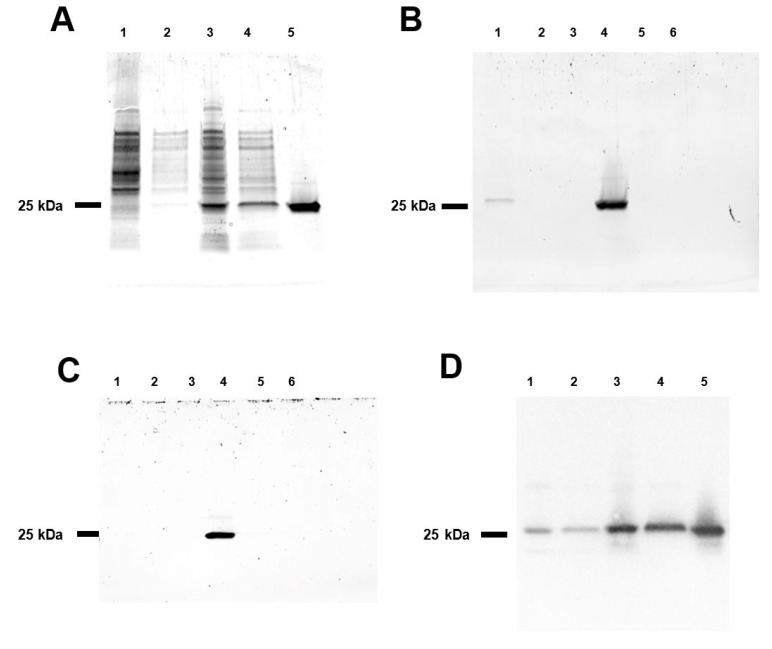
Recombinant Staphylococcal enterotoxin H. (**A**) SDS-PAGE analysis of *E. coli* KRX (pT7-SEH). Lysate (lane 1) and conditioned media (lane 2) from *E. coli* KRX cultured in the absence of rhamnose; lysate (lane 3) and conditioned media (lane 4) from *E. coli* KRX (pT7-SEH) cultured in the presence of rhamnose for 24 h; purified SEH (lane 5). (**B**) HiTrap SP chromatography. Unretained fraction (lane 1), fractions eluted with SP buffer containing 50, 100, 500, 750, 1000 mM NaCl (lanes 2–6, respectively). (**C**) Strong anion exchange chromatography eluted with Q buffer containing 0, 50, 100, 200, 500, or 1000 mM NaCl (lanes 1–6, respectively). (**D**) Western blot of SDS-PAGE loaded identically with panel (**A**) and probed using anti-SEH primary antibodies.

Bacterial expression vectors for generating recombinant SEH have been described in the past [26,27,28]. The pF1AT7 Flexi/*E. coli* KRX system used in this study provides tight repression of SEH expression in the absence of added rhamnose to induce expression of T7 RNA polymerase to offer enhanced biosafety. The addition of rhamnose to *E. coli* KRX (pT7-SEH) induced the extracellular secretion of a protein with an apparent molecular mass of 25 kDa, consistent with the predicted size of the mature form of SEH (Figure 1A). The novel 25 kDa protein was induced strongly in conditioned media collected from the *E. coli* KRX host or *E. coli* (pT7-SEH) cultured in the presence of rhamnose. Strong cation exchange chromatography captured the protein of interest from conditioned culture media adjusted to pH 4.0 and it could be eluted efficiently in 25 mM sodium acetate, 10 µM zinc chloride, pH 4.0 (SP Buffer) containing 500 mM NaCl (Figure 1B). Strong anion exchange chromatography with elution in 25 mM histidine, 10 µM zinc chloride, pH 6.5 (Q Buffer) containing 100 mM NaCl removed remaining contaminants (Figure 1C). Western blots using SEH-specific antibodies identified the protein of interest as the mature form of SEH (Figure 1D).

**Figure 2:**
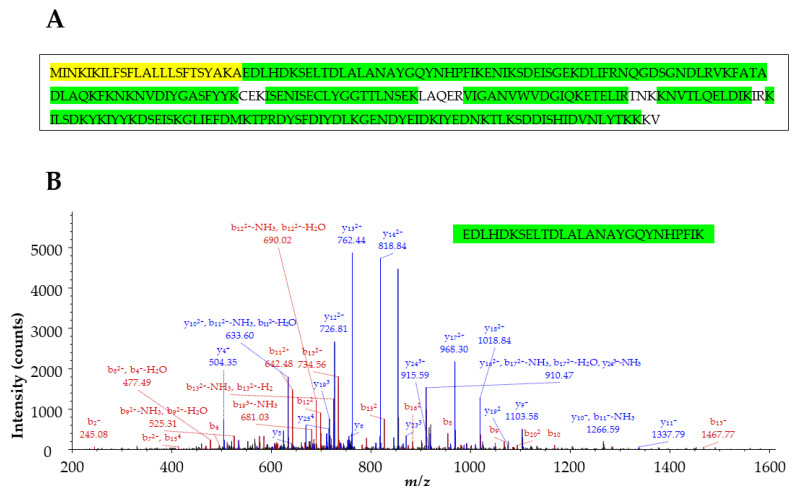
Tandem mass spectrometry (MS/MS) identification of the N-terminal sequence of Staphylococcal enterotoxin H. (**A**) Protein precursor sequence of Staphylococcal enterotoxin H. The signal peptide is highlighted in yellow and was not identified by MS/MS. Peptide sequences identified by MS/MS are highlighted in green. (**B**) MS/MS fragment ions with *b*- (red) and *y*- (blue) series ions matched to the peptide EDLHDKSELTDLALANAYGQYNHPFIK.

To identify the N-terminal peptide of SEH, recombinant SEH purified from culture media was digested with trypsin and the resulting peptides were analyzed by tandem mass spectrometry. The MS/MS data were searched against the *Staphylococcus aureus* proteome sequence database containing both precursor and mature protein sequences for SEH. The top-ranked protein was identified as the mature form of SEH. Figure 2A shows the peptide sequences identified (in green). No peptide sequences were identified from the signal peptide part (in yellow) either as an N-terminal peptide or tryptic peptides cleaved from the signal peptide. EDLHDKSELTDLALANAYGQYNHPFIK and EDLHDK were identified as N-terminal peptide sequences which were only from the mature SEH sequence with mass accuracy of 1.01 ppm and 0.27 ppm, respectively. In addition, the N-terminal ends of these peptides did not result from trypsin digestion. These data suggest that the mature form of SEH was secreted. Additional database search without tryptic restriction confirmed the N-terminal peptides were from the mature form of SEH.

### 2.2. Evaluation of SE Unfolding Using Differential Scanning Calorimetry

**Figure 3:**
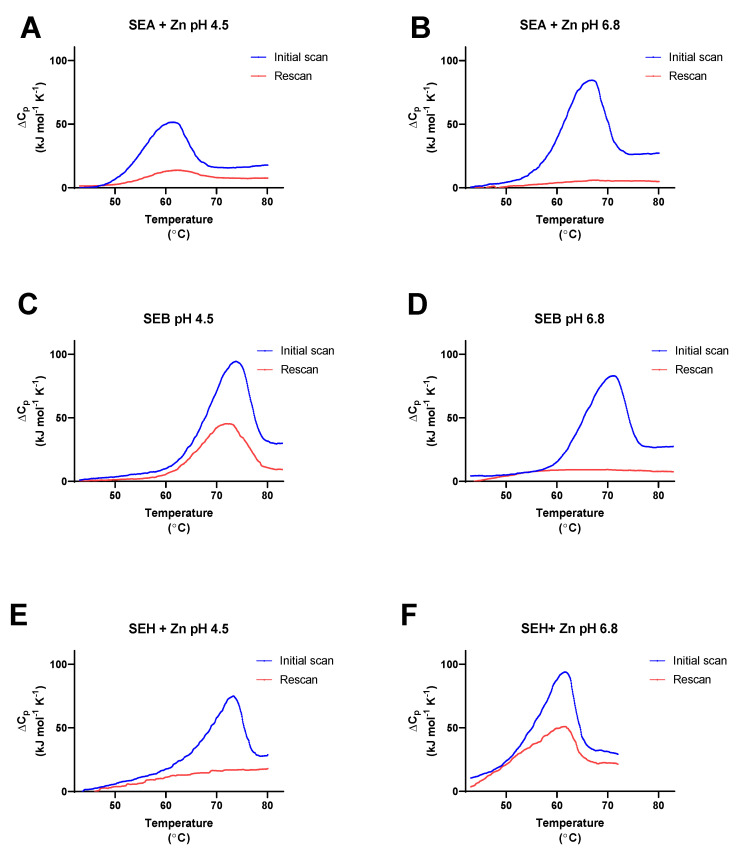
Differential scanning calorimetry of SEA, SEB, and SEH. (**A**) SEA in 25 mM sodium acetate, 10 µM zinc chloride, pH 4.5, (**B**) SEA in 25 mM sodium phosphate, 10 µM zinc chloride, pH 6.8, (**C**) SEB in 25 mM sodium acetate, pH 4.5, (**D**) SEB in 25 mM sodium phosphate, pH 6.8, (**E**) SEH in 25 mM sodium acetate, 10 µM zinc chloride, pH 4.5, and (**F**) SEH in 25 mM sodium phosphate, 10 µM zinc chloride, pH 6.8. SE proteins (0.2–0.3 mg/mL) in the buffers indicated were heated from 22 to 90 °C at 1.0 °C/min (blue traces), cooled from 90 to 22 °C at 1.0 °C/min (not shown), and reheated from 22 to 90 °C (red traces). Data from three replicate trials were averaged for each trace.

**Figure 4:**
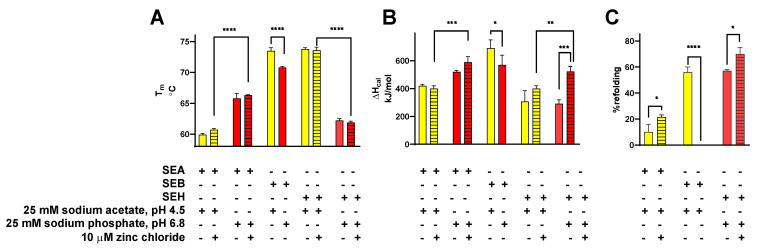
Effects pH and zinc on thermodynamic parameters for SEA, SEB, and SEH. (**A**) T_m_ for heating cycle 1, (**B**) ΔH_cal_ for heating cycle 1, and (**C**) % refolding for complete DSC scans performed in 25 mM sodium acetate (yellow bars) or in 25 mM sodium phosphate (red bars) in the absence (open bars) or presence (striped bars) of 10 µM zinc chloride for the averages from at least three replicate trials. The inclusion or exclusion of reaction components are indicated by “+” and “−“ symbols, respectively. Error bars indicate the standard deviation. *, **, ***, and **** indicate *p* < 0.05, <0.01, <0.001, <0.0001, respectively.

**Figure 5:**
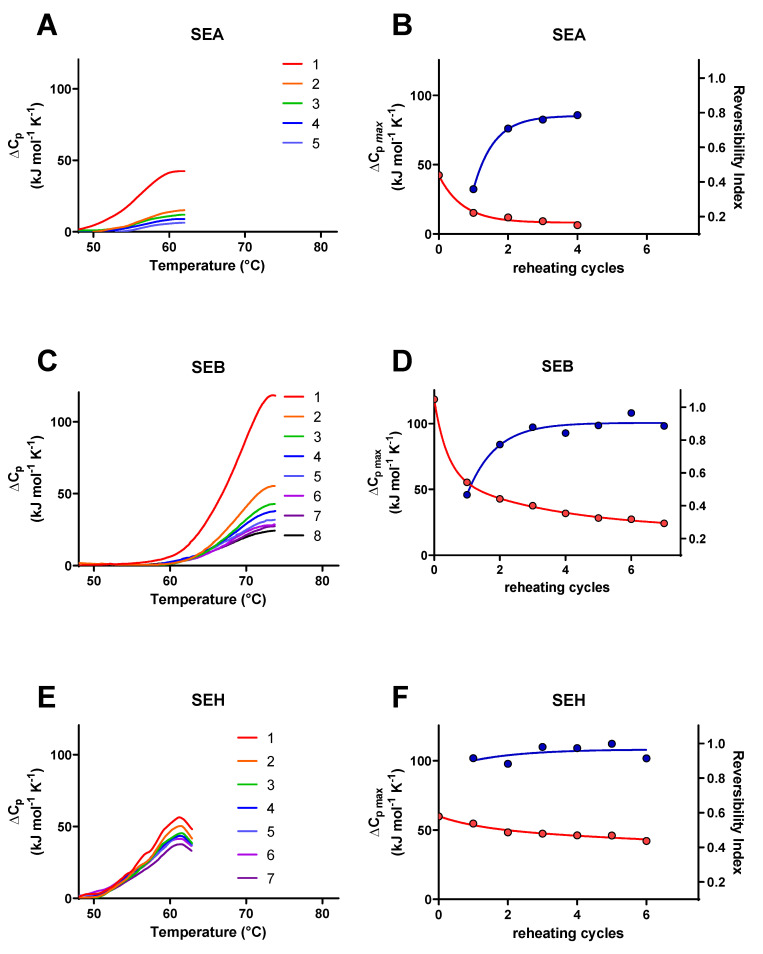
Partial DSC thermograms for SEA, SEB, and SEH. (**A**) SEA in 25 mM sodium acetate, 10 µM zinc chloride, pH 4.5 heated from 22.0 to 62.0 °C, (**C**) SEB in 25 mM sodium acetate, pH 4.5 heated from 22.0 to 73.8 °C, (**E**), SEH in 25 mM sodium phosphate, 10 µM zinc chloride, pH 6.8 heated from 22.0 to 62.8 °C. SE proteins (0.2–0.3 mg/mL) in the buffers indicated were heated at 1.0 °C/min through the temperature ranges given, cooled to 22 °C at 1.0 °C/min (not shown), and reheated through multiple cycles. (**B**,**D**,**F**) Maximum ΔC_p_ values observed for each partial heating cycle for panels A, C, and E are plotted on the left Y-axes (red traces), heating cycle numbers are plotted on the *x*-axes, and the differences in maximum ΔC_p_ values for consecutive cycles are plotted on the right *Y*-axes (blue traces). Data for each curve were fitted to simple exponential decay models.

A well-known characteristic associated with the classical SE proteins is their resistance to thermal inactivation. A biophysical understanding of thermodynamic parameters relevant to SE protein unfolding is less complete, however. DSC was used in this study to measure the observed, or calorimetric, enthalpy (ΔH_cal_), entropy (ΔS_cal_), and melting temperature (T_m_) values for SEA, SEB, and SEH at pH 4.5 and at pH 6.8 scanned from 22 to 90 °C at 1.0 °C per min (Table 1). A cooling cycle from 90 to 22 °C at 1.0 °C per min was included for each sample immediately after the first heating cycle, followed by a second heating cycle conducted using the same parameters as the first scan (Figure 3A–F) to determine % refolding from the ratios of ΔH_cal_ values (Table 1) according to Equation (1) defined in the Materials and Methods (see Section 5.2 Differential Scanning Calorimetry). In addition, theoretical van’t Hoff enthalpy values (ΔH_vH_) were calculated for each transition from the maximum heat capacity (ΔC_pmax_), T_m_, and ΔH_cal_ using Equation (3) (see Section 5.2). A transition following a two-state equilibrium process is indicated by ΔH_cal_/ΔH_vH_ = 1.0, the involvement of unfolding intermediates is indicated by ΔH_cal_/ΔH_vH_ > 1.0, and the presence of oligomeric states and/or cooperative interactions between unfolded domains is indicated by ΔH_cal_/ΔH_vH_ < 1.0. The effects of pH and zinc on T_m_, ΔH_cal_, and % refolding for SEA, SEB, and SEH are presented in Figure 4. Multiple repeated partial DSC scans from 22 °C to T_m_ + 0.5 °C (Figure 5A–F) were used in subsequent experiments to evaluate apparent reversibility indices using Equation (2) (see Section 5.2) under conditions expected to minimize the opportunity for secondary processes to interfere with refolding.

#### 2.2.1. SEA

The presence of endothermic transitions with T_m_ 60.7 ± 0.2 and T_m_ 61.5 ± 0.8 °C for SEA in 25 mM sodium acetate, 10 µM zinc chloride, pH 4.5 buffer for initial and second heating cycles, respectively, indicated that SEA renatured under these conditions, albeit incompletely (Table 1, Figure 3A and Figure 4A,C). The calculated extent of SEA refolding at pH 4.5 in the presence of zinc was 21 ± 2% using the ratio of average ΔH_cal_ values for the first and second heating cycles (Table 1, Figure 3C and Figure 4C). Compared to the behavior of SEA at pH 4.5, a higher T_m_ value (66.3.7 ± 0.1 °C) was observed for the first heating cycles of SEA in 25 mM sodium phosphate, 10 µM zinc chloride, pH 6.8 buffer (Table 1, Figure 3B and Figure 4A), indicative of increased thermal stability at pH 6.8. Similarly, the average ΔH_cal_ for SEA first heating cycles measured at pH 6.8 was higher than that observed at pH 4.5 (590 ± 40 kJ/mol and 400 ± 20 kJ/mol, respectively, Table 1 and Figure 3B, Figure 4B). Despite the enhanced thermal stability noted for SEA at pH 6.8, the absence of endothermic transitions during second heating cycles showed that SEA unfolding was irreversible in the buffer system used. The average ΔS_cal_ value calculated for the first heating cycles of SEA at pH 6.8 (1.7 ± 0.1 kJ/mol K) was greater than for corresponding trials conducted at pH 4.5 (1.2 ± 0.1 kJ/mol K) (Table 1). Furthermore, average ΔS_cal_ values for second heating cycles (ΔS_cal_ 0.17–0.3 kJ/mol K) were lower in every case than those for the corresponding initial heating cycles.

Additional DSC experiments were conducted in the absence of zinc ions using the same buffers to evaluate the effects of metal ion binding on the thermal stability and % refolding of SEA (Table 1). Insignificant effects on T_m_ and ΔH_cal_ were observed for SEA in the absence of zinc in sodium acetate buffer at pH 4.5 for the first heating cycles (Table 1 and Figure 4A,B). However, the decreased ΔH_cal_ observed for SEA in the absence of zinc for its second heating cycle resulted in a proportional reduction in % refolding of SEA from 21 ± 2 in the presence of zinc to 13 ± 4% in its absence at pH 4.5 (Table 1 and Figure 4B,C). Moreover, SEA exhibited a decreased ΔH_cal_ value (520 ± 13 kJ/mol) in the absence of zinc in sodium phosphate buffer at pH 6.8 and an insignificant effect on T_m_ when compared to ΔH_cal_ and T_m_ values for SEA in the presence of zinc, consistent with diminished internal energy stabilizing the tertiary structure of zinc-depleted SEA (Table 1, Figure 4A,B). The absence of zinc resulted in negligible effects on average ΔS_cal_ values for SEA at pH 4.5 or pH 6.8 (Table 1). The first heating cycles for SEA resulted in ΔH_cal_/ΔH_vH_ values between 1.1 and 1.3, consistent with monomeric SEA unfolding from its native to unfolded states with minimal involvement of unfolded intermediates in sodium acetate, pH 4.5 or sodium phosphate, pH 6.8, and in the presence or absence of zinc (Table 1). Lower ΔH_cal_/ΔH_vH_ values (0.1–0.3) observed for the second heating cycles of SEA suggest that most of the SEA was denatured irreversibly after the first heating cycles with greatly decreased concentrations of refolded SEA available for unfolding during the second heating cycles.

#### 2.2.2. SEB

Evidence for the efficient refolding of SEB was apparent in 25 mM sodium acetate buffer, pH 4.5 by endothermic transitions at T_m_ 73.5 ± 0.5 and T_m_ 71.8 ± 0.7 °C for the first and second heating cycles, respectively, (Table 1, Figure 3C, Figure 4A). Robust ΔH_cal_ values, 690 ± 60 and 390 ± 10 kJ/mol, observed for consecutive heating cycles for SEB, resulted in 56 ± 4% refolding (Table 1, Figure 4C). In contrast, no indication of SEB renaturation was detected in 25 mM sodium phosphate, pH 6.8 (Figure 3D). Compared to the results obtained for SEB at pH 4.5, the lower average T_m_ 70.8 ± 0.2 °C and diminished ΔH_cal_ 570 ± 70 kJ/mol observed for the first heating cycles of SEB at pH 6.8 signify lower thermal stability for SEB under these conditions (Table 1, Figure 4A,B). The effects of pH on average ΔS_cal_ values observed for the initial heating cycles of SEB at pH 4.5 and pH 6.8 (2.0 and 1.7 kJ/mol K) were similar in magnitude and the average ΔS_cal_ value for second heating cycles at pH 4.5 (1.1 ± 0.1 kJ/mol K) were significantly lower than for the first heating cycle (2.0 ± 0.2 kJ/mol K) (Table 1). Similar to the case for SEA, the first heating cycles for SEB exhibited ΔH_cal_/ΔH_vH_ values 1.3–1.7, suggesting that SEB unfolded with little evidence for the involvement of intermediates (Table 1). Robust ΔH_cal_/ΔH_vH_ values of 1.0 ± 0.1 for the second heating cycles of SEB in 25 mM sodium acetate, pH 4.5 indicated that most of the protein refolded efficiently.

#### 2.2.3. SEH

Thermal unfolding for SEH in 25 mM sodium acetate, 10 µM zinc chloride, pH 4.5 buffer was found to be irreversible, with an endothermic transition at T_m_ 73.6 ± 0.5 °C for the first heating cycles and none for the second heating cycles (Table 1, Figure 3E). However, endothermic transitions at T_m_ 61.9 ± 0.1 and T_m_ 60.6 ± 1.3 °C detected for SEH in 25 mM sodium phosphate, 10 µM zinc chloride, pH 6.8 buffer for first and second heating cycles, respectively (Table 1, Figure 3F, Figure 4A), showed that SEH refolded with 70 ± 5% efficiency under these conditions (Figure 4C). Although the higher T_m_ noted for SEH at pH 4.5 compared to pH 6.8 indicates greater thermal stability for this isoform under acidic conditions, the average ΔH_cal_ value observed at pH 6.8 (520 ± 40 kJ/mol) compared to pH 4.5 (400 ± 40 kJ/mol) indicate that intermolecular forces stabilizing the tertiary structure of SEH are weaker at pH 4.5 than at pH 6.8 (Figure 4B). No significant effects caused by absence of zinc were observed on average T_m_ values for SEH at pH 4.5 or pH 6.8 (Table 1, Figure 4A). However, the average ΔH_cal_ value for SEH at pH 6.8 in the absence of zinc (280 ± 20 kJ/mol) was significantly lower than in the presence of zinc (520 ± 40 kJ/mol) and the efficiency of SEH refolding was reduced to 56 ± 2% (Table 1, Figure 4B,C). Maximal ΔH_cal_/ΔH_vH_ values for SEH, 1.1 ± 0.1, and 1.3 ± 0.1, were observed for the first and second heating cycles in 25 mM sodium phosphate, 10 µM zinc chloride, pH 6.8 buffer, conditions associated with its optimal refolding (Table 1). Acceptable but lower ΔH_cal_/ΔH_vH_ values were observed for first heating cycles of SEH in this buffer in the absence of zinc (0.7 ± 0.1) or in 25 mM sodium acetate, pH 4.5 in the absence (0.7± 0.2) or presence (0.8 ± 0.1) of zinc.

#### 2.2.4. Consecutive Partial DSC Heating Cycles to Evaluate SE Refolding

Aggregation of unfolded polypeptides during DSC trials can interfere with the efficiency of renaturation, particularly at temperatures above T_m_. The buried hydrophobic domains of a protein in its native state are increasingly exposed to the solvent as a protein unfolds, particularly at temperatures above T_m_. These exposed hydrophobic protein domains are then subject to intramolecular protein–protein interactions that occur in a concentration-dependent manner. The intramolecular condensation of hydrophobic domains is generally accompanied by the entropically-favored release of bound solvent water molecules favoring the thermodynamically irreversible formation of unfolded protein aggregates. Reversing a heating cycle slightly above T_m_ can minimize the probability that aggregation or other potential thermochemical side reactions will reduce the observed extent of protein refolding.

Multiple consecutive partial DSC scans were conducted for SEA, SEB, and SEH using buffer conditions that showed the highest % refolding with complete scans in the initial study, i.e., pH 4.5 for SEA and SEB and pH 6.8 for SEH with the addition of 10 µM zinc for SEA and SEH (Figure 5A,C,E). Observed maximum excess heat capacity values (ΔC_pmax_) declined initially and then became stable with repeated partial heating cycles for all three SE isoforms (Figure 5B,D,F). Reversibility indices calculated from the ratios of ΔC_pmax_ values for consecutive heating cycles stabilized at 0.78, 0.91, and 0.97 for SEA, SEB, and SEH, respectively, between heating cycles 2 and 5. The non-zero ΔC_pmax_ plateaus and the reversibility indices approaching unity are consistent with limited permanent losses of tertiary structure for portions of these SE proteins and with almost fully reversible unfolding for other portions. SEA at pH 4.5 exhibited the greatest loss in ΔC_pmax_ while SEB and SEH at pH 4.5 and pH 6.8, respectively, refolded more efficiently. The reversibility indices calculated from the first and second partial heating cycles (0.358 ± 0.001, 0.467 ± 0.007, and 0.916 ± 0.001) were proportional to the % refolding values observed for first and second complete heating cycles (21 ± 2, 56 ± 4, and 70 ± 5) of SEA, SEB, and SEH, respectively. The consistency between the results for complete and partial heating cycles suggests that protein aggregation or other processes did not introduce large inhibitory effects on the efficiency of SE refolding.

#### 2.2.5. Effects of pH on SEB Refolding and Thermodynamic Properties

**Figure 6:**
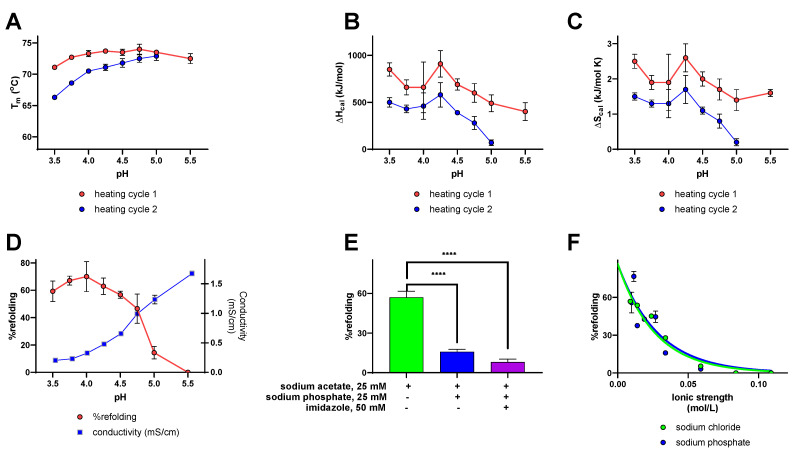
Effects of pH, buffer composition, and ionic strength on the reversible unfolding of SEB. (**A**) T_m_ values for first and second heating cycles of for SEB in 25 mM sodium acetate buffer adjusted to pH 3.5–pH 5.5 with NaOH or HCl, (**B**) ΔH_cal_, (**C**) ΔS_cal_, (**D**) % refolding plotted on the left *Y*-axis (red trace), pH on the *x*-axis, and the conductivity of each buffer on the right *Y*-axis (blue trace). (**E**) % refolding for SEB in 25 mM sodium acetate, pH 4.5 (green bar), 25 mM sodium acetate, 25 mM sodium phosphate, pH 4.5 (blue bar), and 25 mM sodium acetate, 25 mM sodium phosphate, 50 mM imidazole, pH 4.5 (purple bar). The inclusion or exclusion of reaction components are indicated by “+” and “−“ symbols, respectively. (**F**) % refolding for SEB in 25 mM sodium acetate, pH 4.5 with increasing ionic strength adjusted using sodium chloride (green curve) or sodium phosphate (blue curve). Each data point represents the average of three or more trials. Error bars indicate the standard deviation. **** indicates *p* < 0.0001.

SEB was selected for additional study over a wider pH range due to its efficient refolding observed in 25 mM sodium acetate buffer at pH 4.5 and because SEB does not introduce the potential complications of pH-dependent effects on zinc ion binding by SEA or SEH. SEB exhibited maximal thermal stability in 25 mM sodium acetate buffer from pH 4.00 to pH 5.00, with T_m_ 73.3–74.0 °C for the first heating cycles that decreased to 71.1 °C at pH 3.50 and to 72.5 °C at pH 5.50 (Table 2, Figure 6A). The decreases in average T_m_ values for first and second heating cycles were negligible at pH 5.00. But ΔT_m_ values increased steadily to 4.7 ± 0.3 °C at pH 3.50, indicating less efficient recovery of thermal stability for SEB after its first heating cycles under acidic conditions. Calorimetric enthalpy values for the first heating cycles were greatest between pH 3.50 and pH 4.75 (600–910 kJ/mol) and decreased significantly at pH 5.00 to pH 5.50 (490–540 kJ/mol) (Figure 6B). Average ΔH_cal_ values also decreased significantly to a greater extent for second heating cycles from 430–580 kJ/mol at pH 3.50 to pH 4.25 to 0–390 kJ/mol at pH 4.5 to pH 5.50. Similar pH-dependent trends were apparent for ΔS_cal_ values for the first and second heating cycles of SEB; ΔS_cal_ values for the first heating cycle remained higher from pH 3.50 to pH 4.75 (1.7–2.6 kJ/mol K) and decreased to 1.4–1.6 kJ/mol K at pH 5.00 to pH 5.50, while ΔS_cal_ values for the second heating cycles were higher from pH 3.50 to pH 4.25 and decreased significantly at pH 4.50 to pH 5.50 (Figure 6C). SEB underwent more efficient refolding (56–72%) under acidic conditions from pH 3.50 to pH 4.50 that decreased from 47% refolding at pH 4.75 to no observable refolding at pH 5.50 (Figure 6D).

#### 2.2.6. Effects of Buffer Type and Concentration on SEB Refolding and Thermodynamic Properties

Table 2 and Figure 6A show that SEB exhibited minor changes in thermal stability for initial heating cycles in sodium acetate buffers, pH 3.5–pH 5.5, with T_m_ values 71.1–74.0 °C. Calorimetric enthalpy values increased to a maximum at pH 4.25 for first and second heating cycles (Figure 6B) that paralleled maximum calorimetric entropy values at pH 4.25 (Figure 6C). Maximum % refolding for SEB was observed from pH 3.5 to pH 4.50, which then declined to zero refolding at pH 5.5 (Figure 6D). The pH was adjusted in these trials by adding increasing amounts of NaOH with the sum of uncharged acetic acid and its ionized conjugate base held constant at 25 mM in each case. The electrical conductivity of these buffers, an indicator of increasing ionic strength, increased as the pH increased due the introduction of sodium ions and the formation of acetate anions from acetic acid (Figure 6D). Thus, optimal refolding efficiency observed for SEB observed from pH 3.50 to pH 4.50 could be due to increased concentrations of hydronium ions, lower ionic strength, or both. Further trials with SEB conducted in 25 mM sodium citrate buffers adjusted to either pH 4.5 or pH 6.8 provided no evidence of refolding (Table 3), discounting the interpretation that the hydronium ion concentration is exclusively responsible for reversible unfolding observed in acidic acetate buffers. This interpretation was confirmed by a decrease in refolding efficiency from 56 ± 4% observed in 25 mM sodium acetate, pH 4.50 (ionic strength 0.00892 mol/L) to 16 ± 2% refolding by the addition of 25 mM sodium phosphate to the same buffer (ionic strength 0.0339 mol/L) (Figure 6E). The possibility that a phosphate-dependent reduction in refolding efficiency was due to ion pair formation between H_2_PO_4_^−^ anions and protonated histidinyl side chains interfering with the reestablishment of salt bridges present in tertiary structure of SEB was tested by adding imidazole as an analog for the histidine R-group. SEB refolding was reduced further to 8 ± 2% in 25 mM sodium acetate, 25 mM sodium phosphate, 50 mM imidazole, pH 4.50 (ionic strength 0.0499 mol/L) (Figure 6E). The alternative interpretation is that the varying refolding efficiencies observed for SEB in different buffer compositions at pH 4.50 are affected by the contribution of buffer ions to the ionic strength. This effect was evident by the reduction in SEB refolding to 19 ± 1% in 83 mM sodium acetate buffer, pH 4.50 (ionic strength 0.0291 mol/L) (Table 3).

Further trials were conducted to compare concentration-dependent effects of sodium phosphate and sodium chloride on the efficiency of SEB refolding in 25 mM sodium acetate buffer at pH 4.50. Figure 6F shows that similar decreases in SEB refolding efficiency were evident with increasing ionic strength with the addition of sodium phosphate or sodium chloride, after adjustment for the partial ionization of weak acids at pH 4.50. Irreversible unfolding of SEB was observed in all trials in which the ionic strength was adjusted to 75 mM by the addition of either sodium phosphate or sodium chloride.

#### 2.2.7. Effects of pH on the Resistance of Staphylococcal Enterotoxin Biological Activity to Thermal Inactivation

SE proteins exert their superantigen activity through specific antigen-independent crosslinking of T-cell receptors (TCR) embedded in the cell membranes of T-lymphocytes with class II major histocompatibility complex (MHC-II) proteins on the surfaces of antigen presenting cells. TCR/MHC-II ligation stimulates the release of excessive amounts of proinflammatory cytokines, such as IFNG.

**Figure 7:**
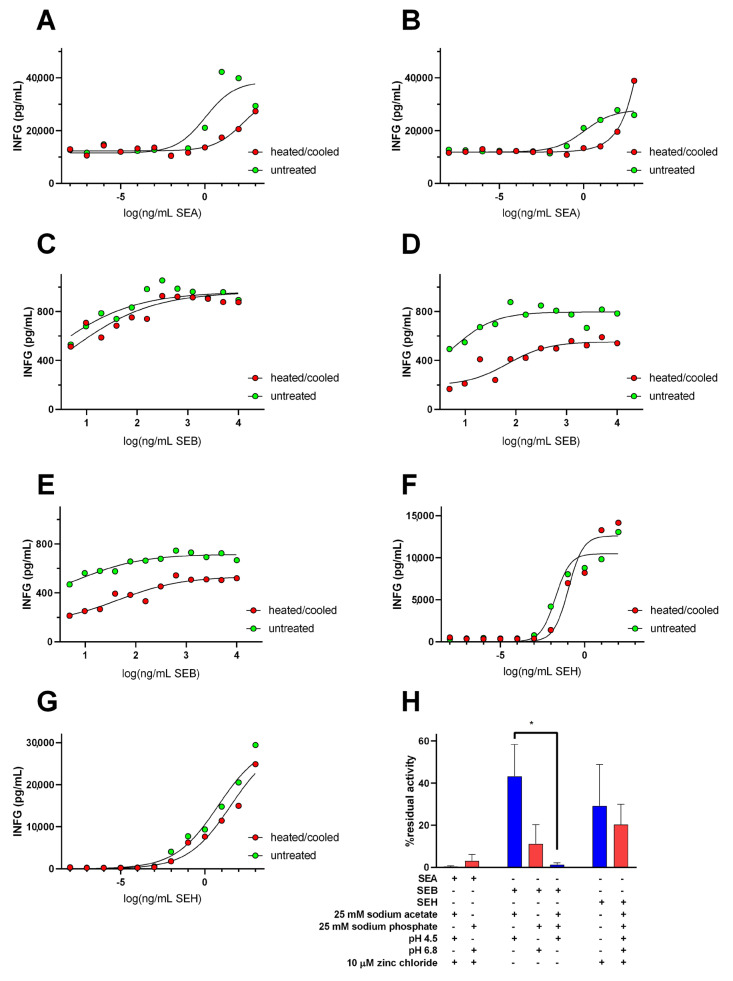
Residual interferon-gamma-inducing activity for SEA, SEB, and SEH after heat treatments in simple buffers. IFNG levels secreted by human peripheral blood mononuclear cells stimulated by untreated or heat-treated: (**A**) SEA in 25 mM sodium acetate, 10 µm zinc chloride buffer, pH 4.5, (**B**) SEA in 25 mM sodium phosphate, 10 µm zinc chloride buffer, pH 6.8, (**C**) SEB in 25 mM sodium acetate buffer, pH 4.5, (**D**) SEB in 25 mM sodium phosphate buffer, pH 6.8, (**E**) SEB in 25 mM sodium acetate, 25 mM sodium phosphate, pH 4.5, (**F**) SEH in 25 mM sodium acetate, 10 µm zinc chloride buffer, pH 4.5, (**G**) SEH in 25 mM sodium phosphate, 10 µm zinc chloride buffer, pH 6.8. Each curve represents the average of at least three trials. (**H**) % residual activities after heat treatment for SEA, SEB, and SEH at pH 4.5 (blue bars) and at pH 6.8 (red bars). The inclusion or exclusion of reaction components are indicated by “+” and “−“ symbols, respectively. Error bars indicate the standard error of the mean and * indicates *p* < 0.05.

The residual Staphylococcal enterotoxin biological activities remaining after heat-treatments were determined from the results of IFNG ELISA studies. SE proteins in the buffers used for DSC experiments were heated and then cooled at rates designed to approximate the heating and cooling program used for DSC. Sodium acetate (25 mM) and sodium phosphate (25 mM) buffers at pH 4.5 and pH 6.8, respectively, were supplemented with 10 µM zinc chloride for trials involving SEA or SEH, but not for trials with SEB. IFNG secretion by SE-treated human peripheral blood mononuclear cells increased in dose-dependent manners in each case (Figure 7A–G). IFNG levels secreted by PBMN cells stimulated by heat-treated or untreated SE proteins were used to determine SE residual activities (Figure 7H).

Although measurable levels of IFNG were secreted by PBMN cells exposed to heat-treated SEA in zinc-containing 25 mM acetate, pH 4.5 or in 25 mM sodium phosphate, pH 6.8 (Figure 7A,B), the average residual biological activities remaining after heat treatments were similar and very low (0.43 ± 0.32% and 3.1 ± 2.9%, respectively, Figure 7H) when compared with the higher residual biological activities exhibited by SEB (Figure 7C,D) and SEH (Figure 7F,G). The % residual activity for SEB after heat treatment at pH 4.5 (43 ± 15%, Figure 7C) appeared to be greater than that detected after heat treatment at pH 6.8 (11.2 ± 9.1%, Figure 7D) as expected from the results of DSC experiments, but the effect of pH on SEB residual activity was not statistically significant. However, the % residual biological activity for SEB decreased significantly to 1.27 ± 0.90% when the protein was heat-treated at pH 4.5 in a 25 mM sodium acetate, 25 mM sodium phosphate buffer (Figure 7E). Similar % residual biological activities were observed for SEH heat-treated at pH 4.5 (29.2 ± 19%, Figure 7F) or at pH 6.8 (20.4 ± 9.6%, Figure 7G).

**Figure 8:**
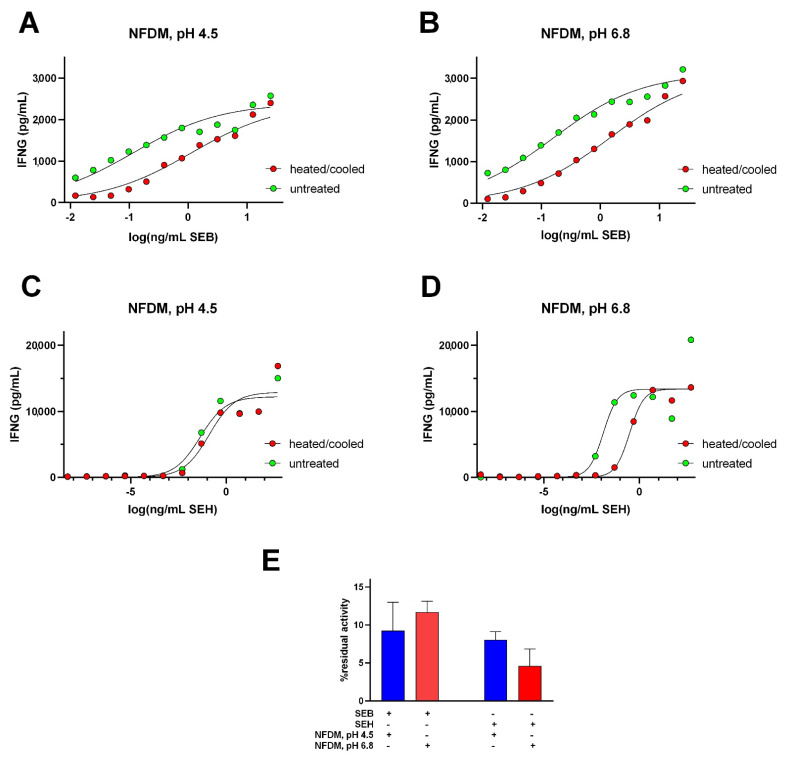
Residual interferon-gamma-inducing activity for SEB and SEH after heat treatments in reconstituted nonfat dried milk adjusted to pH 4.5 or pH 6.8. (**A**) SEB in NFDM, pH 4.5, (**B**) SEB in NFDM, pH 6.8, (**C**) SEH in NFDM, pH 4.5, (**D**) SEH in NFDM, pH 6.8. Each curve represents the average of at least three trials. (**E**) % residual activities for SEB and SEH in NFDM, pH 4.5 (blue bars) and NFDM, pH 6.8 (red bars). The inclusion or exclusion of reaction components are indicated by “+” and “−“ symbols. Error bars represent standard errors of the mean.

The irreversible unfolding observed for SEB at pH 6.8 and for SEH at pH 4.5 in simple buffers led to the prediction of similar effects if these toxins were heat-treated in nonfat dry milk adjusted to those pH values. The lower % refolding observed for SEA by DSC and low % residual biological activity observed for it after heat treatments in simple buffers made it less suitable for further study of its residual activity with heat treatments in NFDM. Comparable decreases in SE-induced IFNG expression were observed for SEB and SEH heated and cooled in NFDM at both pH values (Figure 8A–D), suggesting that a factor present in NFDM other than pH may have contributed to their decreased thermal resistance. Reconstituted NFDM should contain 0.37 mg zinc and 144 mg calcium per 100 mL [29], providing 52 µM zinc and 35.9 mM calcium in the NFDM preparations used in this study. The complex composition of NFDM confounds the direct calculation of its ionic strength and conductivity was used to provide an estimate. Interestingly, the measured conductivity of NFDM adjusted to pH 4.5 with HCl (8.19 ± 0.90 mS/cm) was similar to that of 25 mM sodium acetate, 75 mM NaCl buffer, pH 4.5 (8.28 ± 0.30 mS/cm), conditions leading to irreversible unfolding observed by DSC, and the conductivity of NFDM adjusted to pH 6.8 with NaOH (4.82 ± 0.24 mS/cm) was similar to that of 25 mM sodium acetate, 75 mM sodium phosphate buffer, pH 4.50 (4.92 ± 0.18 mS/cm), conditions which also resulted in irreversible unfolding.

## 3. Discussion

### 3.1. Involvement of SE Proteins in SFP and Their Significance for Food Safety

Current perspectives on the role of the Staphylococcal enterotoxin family in SFP emphasize the sustained importance of these protein toxins in food safety and the hazards they represent for human health [1,3,11,30] and characterizing the involvement of SE proteins in SFP outbreaks remains a significant research topic and public health concern [14,31,32]. Merda et al. [7] performed whole-genome sequence analyses for 244 *S. aureus* isolates associated with SFP outbreaks in Europe from 2005 to 2017 to detect genes for 27 members of the SE protein family (SEA to SEE, SEG to SElX, SElY, SElZ, SEl26, and SEl27) encoded within various mobile genetic elements, including plasmids, prophages, and pathogenicity islands. The results of this study highlight the diversity of SE genes associated with SFP; 71 different SE genetic profiles were identified among the 244 isolates and the notable frequency of sequence variants detected for each SE isoform introduces additional complexity for food safety—which is complicated further by an incomplete understanding of variations in SE gene expression, toxicity, and resistance to heat inactivation in different foods.

*S. aureus* is a significant cause of food-borne disease, causing an estimated 241,000 illnesses per year in the United States although it is likely that this figure is higher due to unreported illnesses [33]. The shedding of *S. aureus* by food handlers harboring these microorganisms is believed to be the most common initiator for SFP, although bovine mastitis may also introduce *S. aureus* into dairy products. Asymptomatic colonization among humans by *S. aureus* can be detected commonly at various anatomic sites, but most notably within the anterior nares, with approximately 20% of the population persistently carrying a single genotype and another 30–50% of the population intermittently infected by different strains [9]. Some relevant examples of SFP outbreaks associated with SE proteins include SEG, SEI, SEM, and SEN detected in sushi and other food items served by a worker harboring the *S. aureus* strain associated with the SFP outbreak [34], closely related *S. argentus* isolates bearing genes for SEB, SEG, SEI, SEM, SEN, SEO, and SElU carried by food handlers in separate SFP outbreaks in 2014 and 2015 [32], a 2003 SFP outbreak in involving SEH-contaminated mashed potatoes prepared with raw milk [8], multiple outbreaks in 1989 resulting from canned mushrooms contaminated with SEA [35], an extensive SFP outbreak in Osaka, Japan that affected more than 10,000 cases who consumed reconstituted powdered skim milk contaminated by low levels of SEA and SEH [12,36] and earlier outbreak involving SEA-contaminated chocolate milk in the US [15].

### 3.2. Resistance of SE Biological Activity to Thermal Inactivation

SEs are highly stable proteins and highly resistant to heat and environmental conditions such as freezing and drying [30]. The underlying role of SE proteins in SFP has prompted studies evaluating their thermal stabilities, their inactivation, and their spontaneous reactivation in foods using a variety of biological activity assays, such as the induction of emesis in cats, dogs, nonhuman primates, or human volunteers, various serological assays, lymphocyte proliferation assays, and the release of inflammatory cytokines [37,38,39,40,41,42] (see Table 4). Our study found that the thermal stability for SEA determined by T_m_ was higher at pH 6.8 in the presence of zinc than in its absence. Additionally, although its thermal stability was lower at pH 4.5 than at pH 6.8, SEA showed evidence of its reversible refolding under acidic conditions, but not at pH 6.8. We found that the thermal stability of SEB and the reversibility of its unfolding at pH 3.5–4.5 were greater than for SEA under any of the experimental conditions tested. Among early studies, Humber et al. [43] found that the emetic activity of SEA in Casamino acids medium at pH 7.8 was less resistant to heating than when the toxin was heated in the same medium at pH 5.3, consistent with our results showing lower thermal stability for SEA in moderately acidic buffers. Similarly, Tatini [44] found that the emetic activity of heat-treated SEA was below the threshold of detection when treated in sodium acetate or sodium phosphate buffers at pH 4.5 to pH 5.5 (ionic strength 0.01) while SEA treated at pH 6.5 to pH 7.5 retained its emetic activity. Jamlang et al. [42] found that heating SEB in 80 mM sodium phosphate, pH 6.4 at 70 °C for 15 min eliminated its emetic activity in dogs, while reheating inactivated SEB for 6 min at 100 °C restored its activity. Schwabe et al. [17] found that the ability of SEA to induce emesis in monkeys was eliminated by heating the toxin for 10 min at 100 °C in 5% gelatin adjusted to pH 4.5. That study also showed that SEA emetic activity was restored by adjusting heat-treated SEA to pH 11 with NaOH followed by immediate neutralization to pH 7.0 with HCl. Enhanced serological detection or the reactivation of SE biological activities following heat treatments has been studied using denaturants such as urea, reheating at different temperatures, and extended incubations at 4 and 25 °C with varying results depending on the toxin, food, assays, and other experimental conditions [16,44,45,46]. The notable thermal stabilities of SE proteins and the significance of their reactivation after heat treatments for food safety is a consistent observation noted in each study.

### 3.3. Structural Features of SE Proteins

The conserved 3-dimensional structures for SE protein family members include several characteristic features; they are monomeric proteins secreted by Staphylococci after the removal of signal peptides from precursor forms to release mature 22–29 kDa simple polypeptide chains composed of an amino-terminal β-barrel domain forming an oligosaccharide/oligonucleotide binding fold (O/B) [51] and a similarly sized carboxyl terminal domain consisting of antiparallel β-strands producing a β-grasp motif that divides SE proteins roughly in half [52]. The SE protein family and the related streptococcal superantigens are subdivided into five groups based on common structural features and biological properties [53]. SEB is placed in Group II, shared with SEC, SEG, SElU, and SElW, proteins having a 10–19 residue cystine loop required for their emetic activities and a low affinity MHC-II α-chain binding site found in the N-terminal O/B fold domain, a feature conserved among all five superantigen groups. The Vβ-TCR binding site is formed by a cleft at the interface between the two protein domains, another feature shared by all five superantigen groups. SEA and SEH are both placed in Group III with SED, SEE, SEJ, SEN, SEO, and SEP based on the presence of a conserved nine residue cystine loop and a zinc-dependent high affinity MHC-II β-chain binding site located in the C-terminal β-grasp domain in addition to the low affinity MHC-II α-chain binding site.

### 3.4. Analysis of Protein Unfolding Using DSC

**Figure 9:**
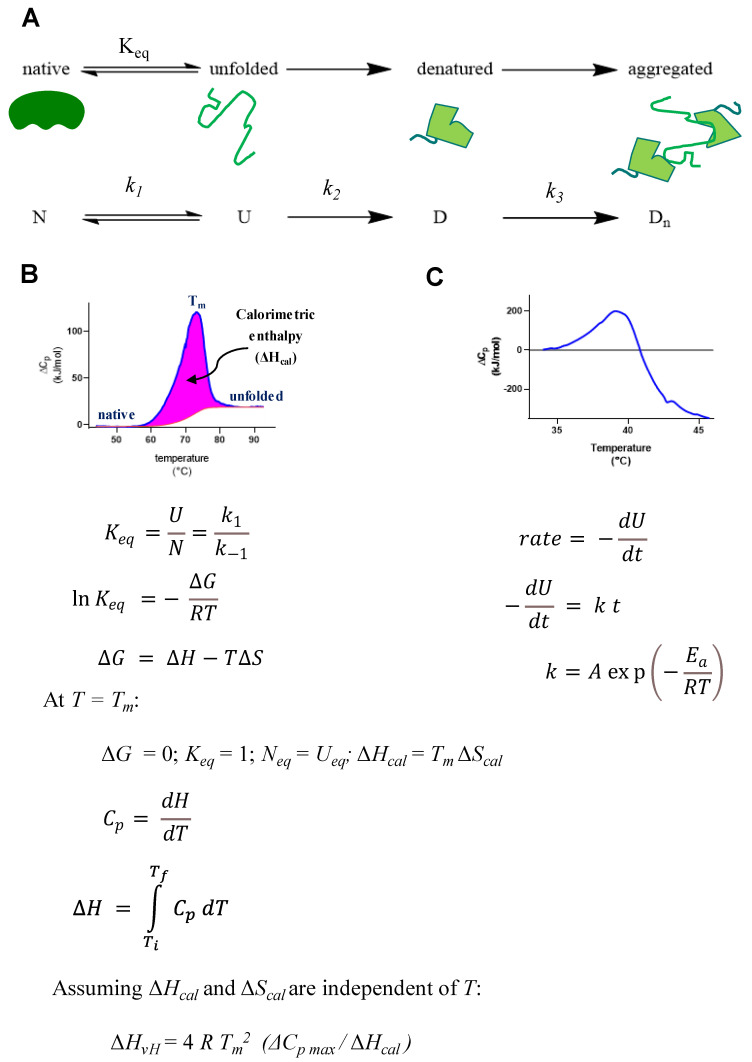
Protein unfolding reactions evaluated by DSC. (**A**) A protein in its native state (N) in equilibrium with unfolded conformations (U) and the concentrations of each is controlled by K_eq_ = U_eq_/N_eq_. Unfolded proteins may undergo irreversible denaturation (D) and the formation of misfolded protein aggregates. (**B**) In the case of reversible protein unfolding, k_2_ and k_3_ are negligible, the rates of protein folding and unfolding become equal at T_m_ in a DSC experiment, U_eq_ = N_eq_, K_eq_ = 1.0, ΔG = 0, and therefore ΔH = TΔS. ΔH for protein unfolding is measured by integrating the observed endothermic change in heat capacity (C_p_) through the transition from native to unfolded states. (**C**) The conformation of an unfolded protein may collapse into misfolded and irreversibly denatured states upon cooling. Intermolecular interactions with other denatured protein molecules result in the aggregation of misfolded protein molecules. The rate of irreversible denaturation is determined by the concentration of the unfolded protein and by a first-order rate constant k_2_ whose magnitude increases with temperature according to the Arrhenius relationship, where E_a_ represents the activation energy barrier. The energetically favored condensation of unfolded or denatured states to form high molecular weight aggregates is controlled by another rate constant, k_3_. Note: thermograms for SEB in 25 mM acetate, pH 4.5 used as the example for (**B**) and ricin in 50 mM sodium phosphate, 50 mM sodium citrate, pH 7.5 for (**C**).

Differential scanning calorimetry is an instrumental method that can be used to determine the change in enthalpy associated with protein unfolding (ΔH_cal_) by integrating the difference in observed heat capacity between a reference cell containing buffer only and a sample cell containing the protein of interest in the reference buffer (ΔC_p_ = C_p_ sample − C_p_ reference) over the temperature range at which the transition from native to unfolded states occurs (Figure 9A). Note that similar symbolism for ΔC_p_ may be used by other authors to indicate the difference between the baseline heat capacity for a protein in its native state prior to the endothermic peak associated with the unfolding process and the (higher) baseline heat capacity of the protein in its fully unfolded state. For this report, however, ΔC_p_ will be used exclusively to refer to the measured difference in heat capacity at a given temperature between the reference and sample cells of the DSC instrument as defined above.

In general, ΔC_p_ reaches its maximum at T_m_ and provides conditions at which the concentrations for the native and unfolded forms of the protein are equal, resulting in K_eq_ = 1, ΔG = 0 and ΔH_cal_ = T_m_ ΔS_cal_ at that temperature (Figure 9B) [19]. The van’t Hoff equation relates K_eq_ determined at one temperature, such as T_m_, to theoretical K_eq_ values at other temperatures, assuming that both ΔH and ΔS are independent of temperature. This assumption is also applied to calculating ΔH_vH_ from T_m_, ΔC_pmax_, and ΔH_cal_ values using Equation (3). Therefore, comparing ΔH_vH_ to ΔH_cal_ tests the suitability of that assumption. An alternative method for calculating ΔH_vH_ substitutes 1/ΔT_1/2_ (where ΔT_1/2_ represents the temperature width at 50% ΔC_pmax_) for ΔC_pmax_/ΔH_cal_ [54,55]. This alternative method can be particularly useful for sharp transitions occurring over narrow temperature ranges typical for some other methods of thermal analysis (e.g., adiabatic scanning calorimetry). Such narrow transitions are not often observed for protein unfolding, where the former equation is applied more commonly [19].

Interestingly, adiabatic scanning calorimetry (ASC) is a method that can utilize extremely low scan rates to maintain thermodynamic equilibrium through the same general temperature ranges used by DSC microcalorimeters to investigate protein unfolding. The very slow scan rates (>0.1 °C/min) provided by ASC are often ideal for investigating phase change transitions with high precision [56]. As indicated in Figure 9C, however, slower scan rates involve prolonged incubations of unfolded proteins above T_m_, which may facilitate secondary reactions (e.g., aggregation) that distort DSC thermograms (*infra vide*). Preliminary DSC scan rate studies are recommended to determine conditions for which observed T_m_ and ΔH_cal_ values are independent of DSC scan rate.

DSC is uniquely suited to study reversible and irreversible protein unfolding. In the simplest case, rescanning a sample after thermal unfolding will reveal whether a protein is able to return to its native state within the temperature and time frame of the experiment to produce an endothermic transition like the one observed during the initial heating cycle. The absence of a transition upon rescanning for a second heating cycle indicates irreversible unfolding under the experimental conditions used. Moreover, the accumulation of unfolded proteins at high temperatures may favor secondary processes that interfere with reversible unfolding, i.e., irreversible denaturation, typically including the formation of protein aggregates that produces a characteristic distortion of a DSC thermogram such as the one for ricin, a different protein, studied in a high ionic strength buffer depicted as an example in Figure 9C.

### 3.5. Effects of pH and Ionic Strength on SE Unfolding

**Figure 10:**
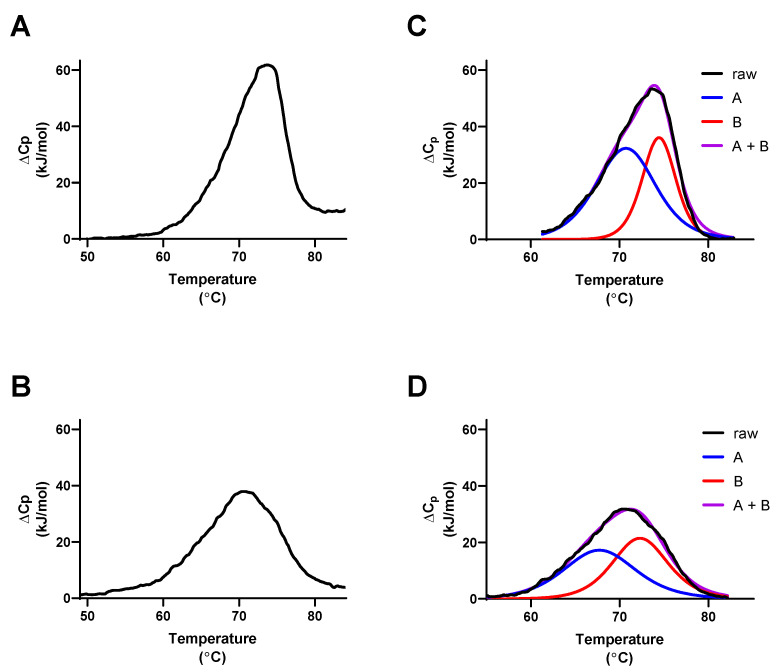
Deconvolution of SEB thermograms. SEB in 25 mM sodium acetate, pH 4.0, (**A**) first heating cycle, (**B**) second heating cycle, (**C**) baseline subtraction and deconvolution of thermogram for first heating cycle, (**D**) baseline subtraction and deconvolution of thermogram for second heating cycle. Data from three replicate trials were averaged for each trace. Black, raw data; blue, lower temperature transition A; red, higher temperature transition B; purple, sum of A + B.

DSC analyses of SE proteins afforded direct measures of T_m_, ΔH_cal_, and ΔS_cal_ under different experimental conditions. The calculation of ΔH_vH_ from T_m_ and ΔH_cal_ measurements and the ΔH_cal_/ΔH_vH_ values provides additional information regarding the nature of thermal unfolding for these proteins. The ΔH_cal_ and ΔH_vH_ values for the initial heating cycles of SEA were similar under all experimental conditions tested, producing ΔH_cal_/ΔH_vH_ values 1.1–1.3 consistent with direct transition between native and unfolded states with minimal involvement of unfolding intermediates. Similar observations were made for the initial heating cycles of SEB (ΔH_cal_/ΔH_vH_ values 1.3–1.7) and SEH (ΔH_cal_/ΔH_vH_ values 0.7–1.1). An understanding of SE tertiary structures, which can be generalized as consisting of an N-terminal β-barrel O/B fold domain and a C-terminal β-grasp domain, each involving about 100 amino acids, provokes the hypothesis that perhaps these domains unfold independently as discernable peaks in a DSC thermogram. Figure 10 depicts the averaged deconvolution of thermograms for the first and second heating cycles for SEB in 25 mM acetate, pH 4.00, selected because these conditions resulted in the highest reversibility of unfolding observed in this study (72 ± 10% refolding). Although the sum of the deconvolved peaks matched the observed ΔC_p_ data well, ΔH_cal_/ΔH_vH_ values calculated for peaks A and B for the thermograms presented were significantly different from 1.0, as they also were for deconvolved peaks A and B determined for SEA, SEB, and SEH thermograms recorded using the other conditions tested (see Supplementary Material Tables S1–S4). Significant involvement of uncharacterized intermediates in the unfolding processes is likely to account for this observation, complicating the interpretation of thermodynamic parameters derived from those analyses.

Yanaka et al. [25] compared protease resistance, HLA-DR binding, cytokine secretion, and thermal stability using DSC for wild-type SEB and several engineered mutants. They reported a T_m_ of 69.7 °C for wild-type SEB in an unspecified buffer and unknown pH with a calorimetric enthalpy of 182.9 kcal/mol (765.3 kJ/mol), values consistent with those observed in our study. Furthermore, we noted significant effects on ΔH_cal_ and % refolding for SEB in sodium acetate buffer, pH 3.5–5.5. Interestingly, significant effects were also noted for ΔT_m_, the difference in T_m_ for sequential heating cycles, which decreased from ΔT_m_ 4.7 ± 0.3 °C at pH 3.5 to ΔT_m_ 0.6 ± 0.7 °C at pH 5.0. The T_m_ values for initial heating cycles remained unchanged while T_m_ values for second heating cycles decreased with increasing acidity as the pH decreased from 5.0 to 3.5. Lower T_m_ values detected for the second heating cycles of SEB under acidic conditions occurred in contrast to the observed trend with increased % refolding as the pH decreased. Together, the results of our study showed that SEB exhibits maximal refolding at pH 4.0, corresponding to greater enthalpies for the first and second heating cycles at that pH interval, and diminished efficiency for refolding as the pH increased to pH 5.5 in sodium acetate buffer or in sodium phosphate at pH 6.8.

We recognized that the ionic strength of the 25 mM sodium acetate buffers used in SEB pH study increased proportionally with increasing pH due to more complete ionization of the acid, reflected by the increase in conductivity from 0.203–1.672 mS/cm from pH 3.5 to pH 5.5. We found that supplementing 25 mM sodium acetate, pH 4.5 (conductivity 0.6563 ± 0.007 mS/cm) buffer with increasing amounts of NaCl resulted in reduced efficiency of SEB refolding and SEB unfolded irreversibly in 25 mM sodium citrate, pH 4.5 (5.16 ± 0.11 mS/cm).

We also considered the possibility that buffer ion–protein binding involving the H_2_PO_4_^−^ anion abundant at pH 4.5 might affect SEB refolding. The concept of dissolved electrolytes interacting with proteins in ways that influence them structurally has extensive biochemical importance. As a recent example, Roberts et al. [57] studied buffer ion effects on protein–protein interactions and found that 10–25 mM phosphate buffer at pH 5 binding to a monoclonal antibody at constant ionic strength, adjusted using NaCl, led to reduced protein–protein repulsion, and that at higher phosphate concentrations the effect was the same as adding more NaCl to screen electrostatic interactions between proteins. In simple terms, smaller multivalent ions with higher charge density and with positive Jones–Dole viscosity coefficients (B) are considered kosmotropes while larger ions with lower charge density and negative Jones–Dole B coefficients are chaotropes [58]. The influences of chaotropic or kosmotropic ions ranked in the Hofmeister series have been reported recently on the stability and reversibility of refolding for lysozyme using DSC [59,60]. These studies showed that kosmotropic cations promoted the efficiency of lysozyme refolding more than chaotropic cations and that kosmotropic anions stabilized lysozyme but favored protein aggregation. Acetate and PO_4_^3−^ anions are considered kosmotropic and the cationic side chains of arginine, lysine, and histidine are chaotropic. Inner sphere ion pair formation is least favored between pairs of oppositely charged kosmotropic and chaotropic ions while ion pair formation is more favorable between ions of similar charge density, e.g., between two kosmotropes, such as Ca^2+^ and PO_4_^3−^ ions resulting in decreased hydration, reduced electrical conductivity, and lower solubility for the ion pair. Trivalent PO_4_^3−^ anions have a greater charge density than divalent HPO_4_^2−^ anions or the monovalent H_2_PO_4_^−^ anions prevalent at pH 4.5, leading to the hypothesis that single layer ion pair formation between H_2_PO_4_^−^ anions and cationic protein residues exposed by SEB unfolding would be more likely than for similar hypothetical interactions with PO_4_^3−^ or HPO_4_^2−^ at higher pH values. We explored this hypothesis by supplementing 25 mM sodium acetate buffer at pH 4.50 with incremental amounts of phosphate and we observed decreasing % refolding for SEB with increasing ionic strength in a manner indistinguishable from the expected ionic shielding effects of adding NaCl, indicating that the formation of specific ion pairs involving H_2_PO_4_^2−^ anions was unlikely to be involved. The addition of imidazole resulted in a further decrease in SEB refolding, consistent with the interpretation that ionic shielding due to increasing ionic strength was responsible for the diminished refolding observed in these experiments.

SEA and SEH possess tetrahedrally coordinated Zn^2+^ binding sites in their C-terminal β-grasp domains that involve a water molecule, His-187, His-225, and Asp-227 residues for SEA (RCSB PDB: 1SXT) and a water molecule, Asn-112, His-205, and Asp-207 residues for SEH (RCSB PDB: 1EWC) [61,62,63,64]. Sundstrom et al. [62] observed high affinity zinc binding (K_d_ 0.3 µM) for SEA in 20 mM HEPES, pH 6.8 at 30 °C using isothermal titration calorimetry in an entropy driven process, with an endothermic ΔH° 5.21 kJ/mol and ΔS° 0.146 kJ/mol K (TΔS—44.2 kJ/mol). Protonation of aspartate (pK_a_ 3.87) and histidine (pK_a_ 6.07) side chains under acidic conditions should interfere with zinc binding by SEA and SEH. Using UV difference spectroscopy, Cavallin et al. [65] found that the addition of 0.1 mM Zn^2+^ caused the T_m_ values for SEA in 20 mM phosphate buffers to increase more dramatically at pH 6.0 and at pH 7.0 than at pH 5.0. Conversely, T_m_ values decreased in the presence of 1 mM EDTA, demonstrating that the thermal stability of SEA is enhanced by zinc binding under neutral or alkaline conditions but shows negligible effects when placed in acidic buffers. Regenthal et al. [66] reported that the T_m_ values for SEA, SEE, and SEH in 20 mM phosphate buffers measured using circular dichroism spectroscopy were higher at pH 6.0 and at pH 7.0 in the presence of 0.1 mM zinc but lower in the presence of 1 mM EDTA than for similar experiments conducted at pH 5.0. Furthermore, protein aggregation was noted for SEA and SEE after consecutive heating and cooling cycles, but not for SEH. Although our DSC experiments found no evidence for SE protein aggregation, SEA and SEH were stabilized by zinc to a greater extent at pH 6.8 compared to pH 4.5 in our study also, consistent with a diminished role for zinc binding under acidic conditions.

The prolonged heat treatments associated with our DSC studies are a constraint introduced by the necessity for thermodynamic equilibrium to be maintained throughout an experiment between folded and unfolded states of the protein of interest. Thus, the heat treatments used for biological assays were not intended to correspond to conditions used for commercial food manufacturing, but to evaluate residual SE activity associated with the extent of refolding observed by DSC. In fact, residual biological activity for heat-treated SEA, SEB, and SEH was detectable in all of our trials involving controlled heating to 90 °C from 22 °C at 1 °C per min and cooling to ambient temperatures, resulting in 36–56 min exposure of SE proteins above T_m_.

## 4. Conclusions

Our study was designed to investigate the reversible thermal unfolding of SEA, SEB, and SEH at pH 6.8 and at pH 4.5; values selected for relevance to raw milk (pH 6.4–pH 6.7) and fermented dairy products (pH 4.4–4.5), respectively. Using DSC, we found that SEB and SEH exhibited similar thermal stabilities at pH 4.5 with T_m_ 73.5–73.6 °C which were significantly greater than that of SEA (T_m_ 60.7 ± 0.12 °C). The thermal stability of SEB (T_m_ 70.8 ± 0.2 °C) was again greater than that of SEA (T_m_ 66.3 ± 0.2 °C) or SEH (T_m_ 61.9 ± 0.1 °C) at pH 6.8. Reversible refolding for SEA, albeit inefficient, was detected for repeated thermal scans at pH 4.5 while irreversible thermal unfolding was observed with repeated scans at pH 6.8. In contrast, SEB provided clear evidence for reversible refolding pH 4.5 but not at pH 6.8. The highest enthalpies for unfolding were noted for SEB (690 ± 60 and 570 ± 70 kJ/mol at pH 4.5 and pH 6.8, respectively), followed by SEA (590 ± 40 kJ/mol) and SEH (520 ± 40 kJ/mol) at pH 6.8. Lower enthalpy and refolding efficiencies were observed for SEA and SEH with zinc depletion, although T_m_ values were largely unaffected. Robust refolding was also observed for SEH at pH 6.8, despite its greater thermal stability evident at pH 4.5. The reversibility index for SEB unfolding at pH 4.5, as calculated from excess heat capacity maxima, increased consistently from 0.50 to 0.95 when comparing six consecutive scans and 0.36 to 0.79 for SEA over five consecutive partial scans at pH 4.5. Similarly, the reversibility indices for SEH were 0.88–0.99 for repetitive partial scans at pH 6.8. The efficiency of refolding for SEB at pH 4.5 was found to be inversely related to the ionic strength using sodium acetate, sodium citrate, sodium phosphate, and sodium chloride. The results of our study clarified differences in the natures of SEA, SEB, and SEH when exposed to heat treatments and demonstrate the utility of DSC for the biophysical characterization of protein toxins relevant to food safety.

## 5. Materials and Methods

### 5.1. Toxins

Laboratory research involving the Staphylococcal enterotoxins requires proper biosafety training, institutional approval and oversight, and appropriate biocontainment practices. This project was conducted in compliance with biosafety guidance from the BMBL [67] and the NIH Guidelines for Research Involving Recombinant or Synthetic Nucleic Acid Molecules [68] and with approval from the FDA Institutional Biosafety Committee and registration with the NIH Office of Science Policy. Research involving Staphylococcal enterotoxins SEA and SEB is regulated by the federal CDC Select Agent Program [69]. Inventories of SEA and SEB were reported annually and were maintained below exempt levels established by Select Agent regulations.

Lyophilized Staphylococcal enterotoxins SEA and SEB were obtained from Toxin Technologies (Sarasota, FL, USA). SEB was reconstituted at 0.5–3 mg/mL in 25 mM sodium acetate, pH 4.5 buffer or 25 mM sodium phosphate, pH 6.8 buffer, as appropriate. SEA was reconstituted in these buffers supplemented with 10 µM zinc chloride.

Controlled expression of recombinant SEH was accomplished using the pFlexi bacterial expression vector system (Promega Corp., Madison, WI, USA). The primers pF1AT7Flexi-751-SEH-fwd and pF1AT7Flexi-751-SEH-rev (Table 5) were designed using the online pFlexi Vector Primer Design Tool (Promega Corporation, Madison, WI, USA). These were used for PCR amplification of the 751 bp SEH precursor open reading frame including its signal peptide sequence for subcloning into the ampicillin-selectable pF1AT7 Flexi expression vector without amino- or carboxyl-fusion peptides. *S. aureus* genomic DNA isolated from NCTR strain 51 (gift of M. Hart, NCTR) provided the template for the SEH precursor open reading frame (Genbank SAU11702) amplified by PCR and introduced into SgfI/PmeI restriction sites of the pF1AT7 Flexi vector using the pFlexi Entry/Transfer kit (Promega Corp) according to the supplier’s instructions, creating a recombinant 3844 bp expression vector product designated pT7-SEH. The structure of pT7-SEH was confirmed by DNA sequence analysis using additional oligonucleotide primers (Table 5) to encode the SEH precursor (AAA19777.1) and then transferred into *E. coli* strain KRX (Promega Corp) for inducible expression of T7 RNA polymerase under the control of the rhamnose promoter.

Expression of recombinant SEH was induced by adding rhamnose (0.1% final concentration) to 15 mL cultures of *E. coli* KRX (pT7-SEH) in LB broth supplemented with 100 µg/mL ampicillin when the optical density at 600 nm reached 0.400–0.500, followed by continued incubation with shaking for 24 h after induction at room temperature. Conditioned media containing secreted SEH was harvested by centrifugation for 20 min at 4500× *g* in sealed rotor buckets 24 h after rhamnose induction. Cell pellets were inactivated by adding 10% bleach. Conditioned media was adjusted to pH 4.0 using HCl, passed through a 0.2 µm sterile filter, applied to a 1 mL HiTrap S column (Cytiva Life Sciences, Marlborough, MA, USA) pre-equilibrated with 25 mM sodium acetate, 10 µM zinc chloride pH 4.0 (SP buffer), and SEH was eluted using a stepwise gradient of 10 mL portions of SP buffer containing 0, 100, 200, 500, 750, and 1000 mM NaCl. Recombinant SEH eluted from the HiTrap SP column in SP buffer with 500 mM NaCl was typically > 95% pure by SDS-PAGE. Further purification of small quantities of SEH was accomplished as needed by adsorption to Pierce Strong Anion Exchange Mini Spin Columns (Thermo Scientific, Waltham, MA, USA) preequilibrated with 25 mM histidine, 10 µM zinc chloride, pH 6.5 (Buffer Q) and eluted with a stepwise gradient of Buffer Q containing 0, 50, 100, 200, 500, or 1000 mM NaCl according to the supplier’s instructions. Purified SEH typically eluted with 100 mM NaCl. The identity of purified SEH was confirmed by Western blotting using affinity-purified anti-SEH rabbit antibodies (Toxin Technology) and KPL Protein Detector Western Blot Kits (Sera-Care Life Sciences, Milford, MA, USA) according to the manufacturer’s instructions and confirmed by mass spectrometry.

Approximately 30 μg of purified recombinant SEH in 25 mM sodium acetate, pH 4.5 or in 25 mM sodium phosphate, pH 6.8 was buffer-exchanged into 70 μL of 50 mM NH_4_HCO_3_, pH 8.0 using Amicon ultra 0.5 mL centrifugal filters (10k cutoff). The protein was then digested by trypsin (Promega, Madison, WI, USA) at 37 °C for 16 h using an enzyme to protein ratio of 1:30 *w*/*w*. The resulting peptides were collected from the filters and lyophilized. The peptides were analyzed by reversed-phase nanoflow LC-tandem mass spectrometry (RP nanoLC-MS/MS) as described previously [70]. Briefly, the peptides were re-dissolved in 300 µL of 0.1% formic acid and 5 µL was injected onto a 180 μm i.d. X 20 mm C18 trap column (100 Å, 5 μm) and separated on a 75 μm i.d. × 150 mm BEH C18 column (130 Å, 1.7 μm, Waters, Milford, MA, USA), which was coupled online to an LTQ-Orbitrap Elite mass spectrometer (Thermo Electron, San Jose, CA, USA). Peptide separation was performed at a flow rate of 0.5 μL/min using a step gradient of 0–42% solvent B (0.1% formic acid in acetonitrile) for 40 min and 42–98% solvent B for 10 min. Both solvents A (0.1% formic acid in water) and B were delivered by a nanoAcquity UPLC system (Waters). The mass spectrometer was operated in a data-dependent mode in which each full MS scan (300–2000 *m*/*z*, acquired in the Orbitrap analyzer) was followed by 15 MS/MS scans (acquired in the ion trap) where the 15 most abundant peptide molecular ions were dynamically selected from the prior MS scan for collision-induced dissociation (CID) using a normalized collision energy of 35%.

A database was constructed to verify the protein sequence of secreted SEH by downloading the *Staphylococcus aureus* proteome sequences from UniProt (http://www.uniprot.org accessed 9 May 2022). In addition to the precursor protein sequence SEH (with the signal peptide) that was already in the database, the sequence of the mature form of this protein (without the signal peptide) was added. The raw MS/MS data were initially searched using the SEQUEST HT running under Proteome Discoverer (version 2.4, Thermo Scientific, San Jose, CA, USA) against the constructed database for the identification of peptides and proteins. Peptide mass tolerance of 10 ppm and fragment ion tolerance of 0.6 Da were set with tryptic specificity allowing two missed cleavages, with dynamic oxidation of Met by addition of one oxygen (+15.9949 Da). An additional database search against the SEH precursor sequence without enzymatic restriction was also performed to identify non-tryptic peptides in addition to the tryptic peptides.

Endotoxin was removed from purified SEH for cell culture experiments by adsorption to 1 mL Pierce High-Capacity Endotoxin Removal Spin Columns (Thermo Scientific) according to the supplier’s instructions. Residual endotoxin levels in SEA, SEB, and SEH samples were less than 0.10 EU/mL as determined using Pierce LAL Chromogenic Endotoxin Quantitation Kits (Thermo Scientific).

Sodium acetate, sodium citrate, sodium phosphate, and other buffers for purification and further experiments were prepared by diluting glacial acetic acid, citric acid, or phosphoric acid into high purity water (18 MΩ/cm) and adjusting the solutions to their final pH values using NaOH. Buffers used for SEA and SEH were supplemented with 10 µM zinc chloride unless otherwise indicated. NaCl or imidazole were added by weight to supplement other buffer formulations as specified. Buffer pH and conductivity were determined using an S47 SevenMulti dual mode meter (Mettler Toledo, Greifensee, Switzerland) with three-point calibrations and the solutions were passed through sterile 0.2 µm filters before use.

The concentrations of SE samples were determined spectrophotometrically after denaturation with urea (5.6 M final concentration). Molar absorptivity coefficients (Table 6) for denatured SEA, SEB, and SEH were calculated based on their amino acid sequences using the Innovagen Peptide Property Calculator online tool (http://pepcalc.com/ppc.php accessed on 22 March 2022).

### 5.2. Differential Scanning Calorimetry

Protein samples were analyzed by differential scanning calorimetry using a Nano DSC Auto microcalorimetry instrument system equipped with an Automated Liquid Sampler (ALS, TA Instruments, Lindon, UT, USA). SE protein samples were equilibrated with appropriate buffers by three cycles of dialysis. Protein concentrations were determined spectrophotometrically, buffers and samples were degassed under reduced pressure using the TA Instruments Degassing Station, and centrifuged for 15 min at 15,000× *g* to remove particles prior to analysis. Three or more DSC trials were conducted for each set of experimental conditions. Buffers paired with SE samples in matching buffers (0.16–0.60 mg/mL SE protein) were placed in the ALS system in sample queues bracketed with appropriate buffers for background subtraction, and lysozyme controls (TA Instruments). Samples and buffers were heated from 22 to 90 °C at 1 °C per minute and cooled from 90 to 22 °C at the same rate with a static nitrogen pressure of ~3 atmospheres pressure under the control of the manufacturer’s DSC Run.exe software (version 4.7.1). Buffer background subtraction and data analyses were performed using NanoAnalyze.exe software (Version 3.12.0). The reversibility of protein unfolding expressed as % refolding was calculated from the ratios of observed, or calorimetric, enthalpy values (ΔH_cal_) for repeated complete scans using Equation (1):

(1) $$\%\;\mathrm{refolding}=100\%\;\left(\frac{\Delta H_{\mathrm{cal}\;\mathrm{heating}\;\mathrm{cycle}\;2}}{\Delta H_{\mathrm{cal}\;\mathrm{heating}\;\mathrm{cycle}\;1}}\right)$$

Alternatively, a reversibility index for SE unfolding was used to evaluate SE protein refolding for repetitive partial scans from 22 °C to immediately past T_m_. The reversibility index was calculated from measured maximum excess heat capacities (ΔC_pmax_) for consecutive heating cycles using Equation (2):

(2) reversibility index=ΔCpmaxcycle iΔCpmaxcycle i−1

Theoretical van’t Hoff enthalpies (ΔH_vH_) were calculated according to Equation (3), where R is the gas constant (8.314 J mol^−1^ K^−1^) and T_m_ is given in kelvins:

(3) ΔHvH=4 R Tm2ΔCp maxΔHcal

### 5.3. Heat Treatments of Staphylococcal Enterotoxins in Sample Buffers or in Reconstituted Nonfat Dry Milk

Sterile buffers used for assays of SE activity were prepared similarly to those used for DSC and passed through sterile 0.2 µm filters before use. Nonfat dry milk (NFDM) obtained from a local market was reconstituted at 100 g/L in high purity molecular biology grade water (Cytiva Life Sciences, Marlborough, MA, USA). The suspension was then stirred for a minimum of 20 min, the pH was adjusted to 4.5 or 6.8 using ~1 M NaOH or HCl, autoclaved at 121 °C for 15 min, and allowed to equilibrate to room temperature overnight. Due to the visible separation of the pH 4.5 NFDM suspension, the transparent layer was carefully removed, centrifuged for 2 min at 17,000× *g*, and the clarified supernatant was used in experiments.

SE samples in pH-adjusted buffers or NFDM were sealed in 96-well V-bottom plates (ProHelix, LabSource, Northlake, IL, USA), heated from 22 to 90 °C and cooled from 90 to 22 °C at 1 °C/min using a Bio-Rad CFX96 Thermal Cycler (Hercules, CA, USA) with a temperature control program designed to mimic the heating and cooling program used for DSC. Identical untreated SE samples were maintained at 22 °C during the heating/cooling cycle. Triplicate trials were repeated under each experimental condition tested.

### 5.4. Enterotoxin Activity Assays

Cryopreserved human peripheral blood mononuclear cells (PBMC, ZenBio, Inc., Durham, NC, USA) were thawed at 37 °C, diluted into RPMI 1640 complete media containing 10% fetal bovine serum and 100 units/mL penicillin and 100 µg/mL streptomycin (Sigma-Aldrich, Burlington, MA, USA), and plated (1.0–2.5 × 105 cells per well in 0.1 mL) in black, polystyrene, clear-bottomed tissue culture-treated 96-well assay plates (Corning, Corning, NY, USA). Cells were allowed to equilibrate at 37 °C in a humidified 5% CO_2_ atmosphere for at least 15 min prior to use in enterotoxin activity assays.

Triplicate sets of serial dilutions of heat-treated SE samples or untreated SE samples were prepared in sterile U-bottom 96-well polystyrene plates using RPMI-1640 complete media as the diluent for experiments involving simple buffers. A diluent containing 1.0 g/L NFDM in RPMI-1640 complete media was substituted for experiments involving SE samples in NFDM. Aliquots (0.1 mL) of serially diluted SE samples were transferred to corresponding wells of 96-well assay plates containing PBMC. Phytohemagglutinin-Leukoagglutinin (PHA) (MP Biomedicals, LLC, Fisher Scientific, Hanover Park, IL, USA) and separate positive control SEs samples were also included. PBMC supernatants were collected after 24–72 h incubation, transferred to a fresh U-bottom 96-well plate, sealed with parafilm, and stored at 4 °C or −20 °C.

### 5.5. Human Interferon-γ ELISA

DuoSet ELISA Development Systems kits, and appropriate accessory kit, for the detection and quantification of human interferon gamma (IFN-γ, INFG) were obtained from R&D Systems, Inc, Minneapolis, MN, USA. The manufacturer’s instructions were slightly modified by adding 1% sodium azide, as a microbial inhibitor, to the plate coating antibody and the calibration curve range was changed to 3.9–2000 pg/mL IFN-γ standard.

### 5.6. Data Analysis

Acid ionization constants and thermodynamic properties for buffer ionization were obtained from Goldberg et al. [71]. Unpaired Student’s T tests were used to compare the means of two groups and one-way or two-way analysis of variance (ANOVA) tests were used to evaluate differences in the means of multiple sample groups (Prism 8.3 software, GraphPad, San Diego, CA, USA). The % residual biological activities remaining in heat-treated Staphylococcal enterotoxin samples were calculated from IFNG ELISA results using Equation (4):

(4) % residual activity=100% EC50 untreatedEC50 heat−treated

where EC_50 (untreated)_ and EC_50 (heat-treated)_ are the effective concentrations of untreated and heat-treated toxin resulting in 50% maximum levels of IFNG detected by ELISA, respectively. Nonlinear regression performed with Prism software was used to fit data from replicate dilutions for IFNG ELISA experiments to Equation (5), where X was the log-transformed SE protein concentration, Y was the concentration of IFNG detected in a trial, Y_min_ and Y_max_ were the minimum and maximum IFNG levels for a given trial, α was the log(EC_50_) value for a series of dilutions, and β was the Hill slope factor.

(5) $$Y={\;Y}_{\min}+\frac{Y_{\max}-{\;Y}_{\min}}{1+10^{\left(\beta \left(\alpha -X\right)\right)}}$$

Differences were considered significant when *p* < 0.05. All experimental trials were repeated at least three times.

### 5.7. Safety

All work with Staphylococcal enterotoxins was conducted with appropriate biocontainment level 2 practices. Experiments with toxins were performed in a certified biological safety cabinet (BSC). Centrifugation or other procedures occurring outside of a BSC were conducted using secondary containment, such as sealed rotors or rotor buckets. Personal protective equipment (lab coats, safety glasses, gloves, etc.) was always used. Glassware and materials exposed to toxins were decontaminated using 10% bleach or by autoclaving at 121 °C for 60 min.

## Figures and Tables

**Table 1:** Thermodynamic properties for SEA, SEB, and SEH unfolding.

Isoform	pH	Zinc (µM)	Heating Cycle	^a^ ΔH_cal_ (kJ/mol)	^b^ ΔH_vH_ (kJ/mol)	$\frac{\Delta H_{cal}}{\Delta H_{vH}}$	^c^ ΔS_cal_ (kJ/mol K)	^d^ T_m_ (°C)	^e^ Percent Refolding
SEA	4.5	0.0	1	419 ± 15	370 ± 2	1.1 ± 0.1	1.3 ± 0.1	59.9 ± 0.2	13 ± 4
			2	55 ± 18	460 ± 80	0.1 ± 0.1	0.17 ± 0.05	60.4 ± 1.9	
		10.0	1	400 ± 20	340 ± 10	1.2 ± 0.1	1.2 ± 0.1	60.7 ± 0.2	21 ± 2
			2	85 ± 6	330 ± 11	0.3 ± 0.1	0.3 ± 0.1	61.5 ± 0.8	
	6.8	0.0	1	520 ± 13	390 ± 20	1.3 ± 0.1	1.5 ± 0.1	65.8 ± 0.8	none
			2	n.d.	n.d.	n.d.	n.d.	n.d.	
		10.0	1	590 ± 40	460 ± 90	1.3 ± 0.2	1.7 ± 0.1	66.3 ± 0.1	none
			2	n.d.	n.d.	n.d.	n.d.	n.d.	
SEB	4.5	0.0	1	690 ± 60	410 ± 10	1.7 ± 0.2	2.0 ± 0.2	73.5 ± 0.5	56 ± 4
			2	390 ± 10	370 ± 10	1.0 ± 0.1	1.1 ± 0.1	71.8 ± 0.7	
	6.8	0.0	1	570 ± 70	430 ± 10	1.3 ± 0.2	1.7 ± 0.2	70.8 ± 0.2	none
			2	n.d.	n.d.	n.d.	n.d.	n.d.	
SEH	4.5	0.0	1	310 ± 80	430 ± 80	0.7 ± 0.2	0.9 ± 0.2	73.8 ± 0.2	none
			2	n.d.	n.d.	n.d.	n.d.	n.d.	
		10.0	1	400 ± 40	490 ± 30	0.8 ± 0.1	1.2 ± 0.3	73.6 ± 0.5	none
			2	n.d.	n.d.	n.d.	n.d.	n.d.	
	6.8	0.0	1	280 ± 20	400 ± 20	0.7 ± 0.1	0.9 ± 0.1	62.1 ± 0.3	56 ± 2
			2	160 ± 20	350 ± 50	0.5 ± 0.1	0.5 ± 0.1	62.2 ± 0.4	
		10.0	1	520 ± 40	480 ± 80	1.1 ± 0.1	1.6 ± 0.1	61.9 ± 0.1	70 ± 5
			2	370 ± 20	290 ± 10	1.3 ± 0.1	1.1 ± 0.1	60.6 ± 1.3	

^a^ ΔH_cal_, calorimetric enthalpy (kJ/mol); ^b^ ΔH_vH_, van’t Hoff enthalpy (kJ/mol), ^c^ ΔS_cal_, calorimetric entropy (kJ/mol K); ^d^ T_m_, thermal transition midpoint; ^e^ percent refolding = ΔH_cal_ rescan/ΔH_cal_ initial scan * (100%). Thermal scans were performed from 22 to 90 °C at 1.0 °C/min. Three or more trials were conducted for each set of conditions. n.d., not detected.

**Table 2:** Effect of pH on thermodynamic properties of SEB unfolding.

pH	Heating Cycle	ΔH_cal_ (kJ/mol)	ΔH_vH_ (kJ/mol)	$\frac{\Delta H_{cal}}{\Delta H_{vH}}$	ΔS_cal_ (kJ/mol K)	T_m_ (°C)	^a^ ΔT_m_ (°C)	Percent Refolding
3.50	1	850 ± 70	530 ± 30	1.6 ± 0.2	2.5 ± 0.2	71.1 ± 0.2	4.7 ± 0.3	59 ± 7
	2	500 ± 50	330 ± 30	1.5 ± 0.2	1.5 ± 0.1	66.3 ± 0.3		
3.75	1	660 ± 80	500 ± 20	1.3 ± 0.1	1.9 ± 0.2	72.7 ± 0.3	4.1 ± 0.3	66 ± 2
	2	430 ± 40	360 ± 10	1.2 ± 0.1	1.3 ± 0.1	68.6 ± 0.2		
4.00	1	660 ± 270	460 ± 20	1.4 ± 0.6	1.9 ± 0.8	73.3 ± 0.5	2.8 ± 0.5	72 ± 10
	2	460 ± 140	360 ± 10	1.3 ± 0.3	1.3 ± 0.4	70.5 ± 0.3		
4.25	1	910 ± 140	450 ± 10	2.0 ± 0.3	2.6 ± 0.4	73.7 ± 0.1	2.5 ± 0.4	63 ± 6
	2	580 ± 130	380 ± 10	1.5 ± 0.3	1.7 ± 0.4	71.1 ± 0.5		
4.50	1	690 ± 60	410 ± 10	1.7 ± 0.2	2.0 ± 0.2	73.5 ± 0.5	1.7 ± 0.8	56 ± 4
	2	390 ± 10	370 ± 10	1.0 ± 0.1	1.1 ± 0.1	71.8 ± 0.7		
4.75	1	600 ± 100	440 ± 10	1.4 ± 0.2	1.7 ± 0.3	74.0 ± 0.8	1.5 ± 1.0	47 ± 10
	2	280 ± 70	400 ± 20	0.7 ± 0.2	0.8 ± 0.2	72.5 ± 0.6		
5.00	1	490 ± 90	450 ± 20	1.1 ± 0.2	1.4 ± 0.3	73.5 ± 0.2	0.6 ± 0.7	14 ± 5
	2	70 ±30	410 ± 50	0.2 ± 0.1	0.2 ± 0.1	72.9 ± 0.7		
5.50	1	540 ± 40	420 ± 20	1.3 ± 0.2	1.6 ± 0.1	72.5 ± 0.8	none	none
	2	n.d.	n.d.	n.d.	n.d.	n.d.		

^a^ ΔT_m_ = T_m_ for heating cycle 1—T_m_ for heating cycle 2.

**Table 3:** Effects of acetate, citrate, phosphate buffers on thermodynamic properties of SEB unfolding.

^a^ Buffer	pH	Heating Cycle	ΔH_cal_ (kJ/mol)	ΔH_vH_ (kJ/mol)	$\frac{\Delta H_{cal}}{\Delta H_{vH}}$	ΔS_cal_ (kJ/mol K)	T_m_ (°C)	ΔT_m_ (°C)	Percent Refolding
25 mM NaOAc	4.50	1	690 ± 60	410 ± 10	1.7 ± 0.2	2.0 ± 0.2	73.5 ± 0.5	1.7 ± 0.8	56 ± 4
		2	390 ± 10	370 ± 10	1.0 ± 0.1	1.1 ± 0.1	71.8 ± 0.7		
25 mM NaP_i_	6.80	1	570 ± 70	430 ± 10	1.3 ± 0.2	1.7 ± 0.2	70.8 ± 0.2	n.d.	n.d.
		2	n.d.	n.d.	n.d.	n.d.	n.d.		
25 mM NaCit	4.50	1	420 ± 5	500 ± 30	0.8 ± 0.1	1.2 ± 0.2	72.0 ± 0.1	n.d.	n.d.
		2	n.d.	n.d.	n.d.	n.d.	n.d.		
25 mM NaCit	6.80	1	510 ± 60	420 ± 30	1.2 ± 0.2	1.5 ± 0.2	69.3 ± 0.7	n.d.	n.d.
		2	n.d.	n.d.	n.d.	n.d.	n.d.		
25 mM NaOAc, 25 mM NaP_i_	4.50	1	560 ± 40	530 ± 90	1.1 ± 0.1	1.6 ± 0.1	73.6 ± 0.6	2.4 ± 0.8	16 ± 2
		2	90 ± 10	340 ± 80	0.3 ± 0.1	0.26 ± 0.03	71.2 ± 0.6		
25 mM NaOAc, 25 mM NaP_i_, 50 mM Im	4.50	1	540 ± 80	490 ± 10	1.1 ± 0.2	1.6 ± 0.2	73.5 ± 0.1	1.9 ± 1.5	8 ± 2
		2	43 ± 8	350 ± 60	0.1 ± 0.1	0.13± 0.02	71.6 ± 1.5		
83 mM NaOAc	4.50	1	650 ± 11	460 ± 10	1.4 ± 0.2	1.9 ± 0.1	73.4 ± 0.4	1.9 ± 0.5	19 ± 1
		2	122 ± 8	390 ± 10	0.3 ± 0.1	0.35 ± 0.02	71.5 ± 0.4		

^a^ buffers: OAc, acetate; P_i_, phosphate; Cit, citrate: Im, imidazole.

**Table 4:** Selected thermal stability and reactivation studies for Staphylococcal enterotoxin biological activities.

Toxins and Sample Treatment	Assay System	Results	Reference
30 µg/mL SEB in 40 mM barbital buffer, pH 7.2, heated at 96–126.7 °C for 12–103 min.	DIDA, emesis (cats)	SEB z-value in veronal (barbital) buffer 32.4 °C, emetic activity inactivated by 115.6 °C for 32.5 min.	[47]
Crude SEA heated at 212–250 °F for 2–100 min.	Emesis (monkeys), emesis (cats)	SEA z-value in culture media 48 °F (emesis in cats), F_250_^49^ 11 min (emesis in cats), F_250_^40^ 8 min (emesis in monkeys)	[40]
30 µg/mL SEB in raw milk heated at 210–260 °F	DIDA	Inactivation times 134.2–12.1 min, D-values 68.5–6.2 min, SEB z-value in milk 46.6 °F.	[48]
100 µg/mL SEB in sodium acetate, pH 4.5 or sodium phosphate, pH 6.4 adjusted to 0.02–1.0 ionic strength heated at 70–100 °C for 0–32 min.	SIDA, emesis (dogs)	SEB resistance to heat inactivation was greater at pH 4.5 than at pH 6.8.	[42]
5–60 µg/mL SEA in beef bouillon, pH 6.2 or PBS, pH 7.2 heated at 100–121.1 °C for 0–200 min.	SIDA	SEA z-value in beef bouillon 27.8 °C	[49]
5–400 ng/kg SEA 2% gelatin/saline or 0.3% peptone, pH 7.0 heated at 100 °C for 25.4 min	Emesis (humans)	SEA-induced emesis resisted heat treatment (6/6). Clinical symptoms from heat-treated SEA appeared more severe than for untreated SEA.	[41]
7 µg/mL SEA in Casamino acids (pH 5.3 or 7.8) or 5 µg/mL SEA in beef bouillon (pH 5.3 or 6.2) heated at 212–250 °F for 1–160 min.	SIDA, emesis (monkeys)	Emetic activity of SEA in Casamino acids, pH 5.3 was eliminated by heating 1 min at 212–250 °F but inactivation required 8–50 min at pH 7.8. SEA z-value in beef bouillon 55 °F.	[43]
0.008–5.0 µg/mL SEB in milk and liquid foods heated at 80–100 °C for 0–5 min. Reactivation at 4 °C and 25 °C for 24 h.	SIDA, DIDA, RIA	SEB in milk, pH 6.4 decreased to 9% of original after 5 min at 100 °C. SEB in buttermilk, pH 4.5 decreased to 26% after 5 min at 100 °C.	[46]
0.1 µg/mL SEA, SEB, or SEC in PBS, pH 7.4 heated at 80, 100, or 120 °C for up to 180 min.	RIA	Heat resistance (descending order): SEC > SEB > SEA.	[50]
5 µg/mL SEA or 1 µg/mL SEB or SEC in milk or food extracts at pH 4.0, 5.5, or 7.0 heated at 80 or 100 °C for 10 min. Reactivation tested with heat and with pH 11 treatment.	ELISA, emesis (monkeys)	Lower recovery of SEA in milk at pH 4.0 and 5.5 than pH 7.0 (29%). Higher recovery of SEB in milk at pH 4.0 than at pH 5.5 or 7.0. Enhanced recovery of SEA and SEB with high pH treatment after heating than with reheating. SEA emetic activity in foods at pH 4.5 or 5.0 restored by high pH after heating.	[17]
0.2–1.0 µg/g SEA in mushrooms or in meat samples heated at 121.1 °C for 0–15 min ± treatment with 6 M urea post-heating	RPLA, ELISA	0.8–2.4% SEA recovered after heating 5–15 min at 121.1 °C. Urea treatment did not improve recovery.	[45]

D-value, the decimal reduction time, is the time at a given temperature to reduce toxin level by 90%. F_T_^R^ value, the thermal reduction time, is the time at temperature T required to reduce toxin to level R. Z-value, temperature shift necessary to reduce a D-value by 90%. DIDA, double gel immunodiffusion assay; RPLA, reversed passive latex agglutination assay; RIA, radioimmunoassay; SIDA, single gel immunodiffusion assay.

**Table 5:** Oligonucleotide primer sequences.

Primer	Sequence (5′-3′)
pF1AT7Flexi-751-SEH-fwd	GCGTGCGATCGCCATGATTAATAAAATTAAAATATTATTTTCGT
pF1AT7Flexi-751-SEH-rev	AACTGTTTAAACTTATACTTTTTTCTTAGTATATAGATT
pFlexi-F	CGGATGGCCTTTTTGCGTTTCTA
pFlexi-R	CTTCCTTTCGGGCTTTGTTAG
SEH-F-354	TGAATGTCTATATGGAGGTACAACA
SEH-F-554	GCGAAATAAGTAAAGGTCTAATTGAA
SEH-R-239	TCATTGCCACTATCACCTTGA
SEH-R-639	ATTTTCTCCTTTTAAATCATAAATGTC

**Table 6:** Staphylococcal enterotoxin properties.

	SEA	SEB	SEH
**Accession**	AAA26681.1	AAW37877.1	AAA19777.1
**3D structure**	1SXT	3SEB	1EWC
**Amino acid residues**	233	239	217
**Molecular mass (kDa)**	27.091	28.366	25.141
**Disulfide links**	C96–C106	C93–C113	C82–C92
**Zinc contact residues**	H187, H225, A227	None	N112, H206, D208
**Isoelectric point (pI)**	6.76	8.91	4.89
**Molar absorptivity (M^−1^ cm^−1^)**	34,540	32,690	23,730

## Data Availability

All data will be placed in the NCTR FDA Archives and can be made available upon request.

## Supplementary Materials

The following supporting information can be downloaded at: https://www.mdpi.com/article/10.3390/toxins14080554/s1, Table S1: Deconvolution for SEA endothermic unfolding; Table S2: Deconvolution for SEB endothermic unfolding; Table S3: Deconvolution for SEB pH study endothermic unfolding; and Table S4: Deconvolution for SEH endothermic unfolding.

## References

[1] Benkerroum N. (2018). Staphylococcal enterotoxins and enterotoxin-like toxins with special reference to dairy products: An overview. Crit. Rev. Food Sci. Nutr..

[2] Krakauer T. (2019). Staphylococcal Superantigens: Pyrogenic Toxins Induce Toxic Shock. Toxins.

[3] Fisher E.L., Otto M., Cheung G.Y.C. (2018). Basis of Virulence in Enterotoxin-Mediated Staphylococcal Food Poisoning. Front. Microbiol..

[4] Etter D., Schelin J., Schuppler M., Johler S. (2020). Staphylococcal Enterotoxin C-An Update on SEC Variants, Their Structure and Properties, and Their Role in Foodborne Intoxications. Toxins.

[5] Huvenne W., Hellings P.W., Bachert C. (2013). Role of staphylococcal superantigens in airway disease. Int. Arch. Allergy Immunol..

[6] Umeda K., Nakamura H., Yamamoto K., Nishina N., Yasufuku K., Hirai Y., Hirayama T., Goto K., Hase A., Ogasawara J. (2017). Molecular and epidemiological characterization of staphylococcal foodborne outbreak of *Staphylococcus aureus* harboring seg, sei, sem, sen, seo, and selu genes without production of classical enterotoxins. Int. J. Food Microbiol..

[7] Merda D., Felten A., Vingadassalon N., Denayer S., Titouche Y., Decastelli L., Hickey B., Kourtis C., Daskalov H., Mistou M.Y. (2020). NAuRA: Genomic Tool to Identify Staphylococcal Enterotoxins in *Staphylococcus aureus* Strains Responsible for FoodBorne Outbreaks. Front. Microbiol..

[8] Jorgensen H.J., Mathisen T., Lovseth A., Omoe K., Qvale K.S., Loncarevic S. (2005). An outbreak of staphylococcal food poisoning caused by enterotoxin H in mashed potato made with raw milk. FEMS Microbiol. Lett..

[9] Bencardino D., Amagliani G., Brandi G. (2021). Carriage of *Staphylococcus aureus* among food handlers: An ongoing challenge in public health. Food Control.

[10] Argudin M.A., Mendoza M.C., Rodicio M.R. (2010). Food poisoning and *Staphylococcus aureus* enterotoxins. Toxins.

[11] Abril A.G., Villa T.G., Barros-Velazquez J., Canas B., Sanchez-Perez A., Calo-Mata P., Carrera M. (2020). *Staphylococcus aureus* Exotoxins and Their Detection in the Dairy Industry and Mastitis. Toxins.

[12] Asao T., Kumeda Y., Kawai T., Shibata T., Oda H., Haruki K., Nakazawa H., Kozaki S. (2003). An extensive outbreak of staphylococcal food poisoning due to low-fat milk in Japan: Estimation of enterotoxin A in the incriminated milk and powdered skim milk. Epidemiol. Infect..

[13] Kerouanton A., Hennekinne J.A., Letertre C., Petit L., Chesneau O., Brisabois A., De Buyser M.L. (2007). Characterization of *Staphylococcus aureus* strains associated with food poisoning outbreaks in France. Int. J. Food Microbiol..

[14] Ciupescu L.M., Auvray F., Nicorescu I.M., Meheut T., Ciupescu V., Lardeux A.L., Tanasuica R., Hennekinne J.A. (2018). Characterization of *Staphylococcus aureus* strains and evidence for the involvement of non-classical enterotoxin genes in food poisoning outbreaks. FEMS Microbiol. Lett..

[15] Evenson M.L., Hinds M.W., Bernstein R.S., Bergdoll M.S. (1988). Estimation of human dose of staphylococcal enterotoxin A from a large outbreak of staphylococcal food poisoning involving chocolate milk. Int. J. Food Microbiol..

[16] Fung D.Y., Steinberg D.H., Miller R.D., Kurantnick M.J., Murphy T.F. (1973). Thermal inactivation of staphylococcal enterotoxins B and C. Appl. Microbiol..

[17] Schwabe M., Notermans S., Boot R., Tatini S.R., Kramer J. (1990). Inactivation of staphylococcal enterotoxins by heat and reactivation by high pH treatment. Int. J. Food Microbiol..

[18] Spink C.H. (2008). Differential scanning calorimetry. Methods Cell Biol..

[19] Hinz H.J., Schwarz F.P. (2001). Measurement and analysis of results obtained on biological substances with differential scanning calorimetry. Pure Appl. Chem..

[20] Vargas-Uribe M., Rodnin M.V., Ojemalm K., Holgado A., Kyrychenko A., Nilsson I., Posokhov Y.O., Makhatadze G., von Heijne G., Ladokhin A.S. (2015). Thermodynamics of Membrane Insertion and Refolding of the Diphtheria Toxin T-Domain. J. Membr. Biol..

[21] Mariutti R.B., Souza T.A., Ullah A., Caruso I.P., de Moraes F.R., Zanphorlin L.M., Tartaglia N.R., Seyffert N., Azevedo V.A., Le Loir Y. (2015). Crystal structure of *Staphylococcus aureus* exfoliative toxin D-like protein: Structural basis for the high specificity of exfoliative toxins. Biochem. Biophys. Res. Commun..

[22] Pina D.G., Gomez J., Villar E., Johannes L., Shnyrov V.L. (2003). Thermodynamic analysis of the structural stability of the shiga toxin B-subunit. Biochemistry.

[23] Krupakar J., Swaminathan C.P., Das P.K., Surolia A., Podder S.K. (1999). Calorimetric studies on the stability of the ribosome-inactivating protein abrin II: Effects of pH and ligand binding. Biochem. J..

[24] Zhang Z., Triplett O.A., Nguyen K.T., Melchior W.B., Taylor K., Jackson L.S., Tolleson W.H. (2013). Thermal inactivation reaction rates for ricin are influenced by pH and carbohydrates. Food Chem. Toxicol..

[25] Yanaka S., Kudou M., Tanaka Y., Sasaki T., Takemoto S., Sakata A., Hattori Y., Koshi T., Futaki S., Tsumoto K. (2010). Contribution of the flexible loop region to the function of staphylococcal enterotoxin B. Protein Eng. Des. Sel. (PEDS).

[26] Ren K.Y., Bannan J.D., Pancholi V., Cheung A.L., Robbins J.C., Fischetti V.A., Zabriskie J.B. (1994). Characterization and biological properties of a new staphylococcal exotoxin. J. Exp. Med..

[27] Nilsson H., Bjork P., Dohlsten M., Antonsson P. (1999). Staphylococcal enterotoxin H displays unique MHC class II-binding properties. J. Immunol..

[28] Rudenko N.V., Karatovskaya A.P., Noskov A.N., Shepelyakovskaya A.O., Shchannikova M.P., Loskutova I.V., Artyemieva O.A., Nikanova D.A., Gladyr E.A., Brovko F.A. (2018). Immunochemical assay with monoclonal antibodies for detection of staphylococcal enterotoxin H. J. Food Drug Anal..

[29] Food Surveys Research Group (USDA) (2017). What’s in the Foods You Eat Search Tool, 2017–2018.

[30] Hennekinne J.A., De Buyser M.L., Dragacci S. (2012). *Staphylococcus aureus* and its food poisoning toxins: Characterization and outbreak investigation. FEMS Microbiol. Rev..

[31] Bastos C.P., Bassani M.T., Mata M.M., Lopes G.V., da Silva W.P. (2017). Prevalence and expression of staphylococcal enterotoxin genes in *Staphylococcus aureus* isolated from food poisoning outbreaks. Can. J. Microbiol..

[32] Wakabayashi Y., Umeda K., Yonogi S., Nakamura H., Yamamoto K., Kumeda Y., Kawatsu K. (2018). Staphylococcal food poisoning caused by *Staphylococcus argenteus* harboring staphylococcal enterotoxin genes. Int. J. Food Microbiol..

[33] Scallan E., Hoekstra R.M., Angulo F.J., Tauxe R.V., Widdowson M.A., Roy S.L., Jones J.L., Griffin P.M. (2011). Foodborne illness acquired in the United States—major pathogens. Emerg. Infect. Dis.

[34] Umeda K., Ono H.K., Wada T., Motooka D., Nakamura S., Nakamura H., Hu D.L. (2021). High production of egc2-related staphylococcal enterotoxins caused a food poisoning outbreak. Int. J. Food Microbiol..

[35] Levine W.C., Bennett R.W., Choi Y., Henning K.J., Rager J.R., Hendricks K.A., Hopkins D.P., Gunn R.A., Griffin P.M. (1996). Staphylococcal food poisoning caused by imported canned mushrooms. J. Infect. Dis..

[36] Ikeda T., Tamate N., Yamaguchi K., Makino S. (2005). Mass outbreak of food poisoning disease caused by small amounts of staphylococcal enterotoxins A and H. Appl. Environ. Microbiol..

[37] Rasooly R., Do P.M., Friedman M. (2010). Inhibition of biological activity of staphylococcal enterotoxin A (SEA) by apple juice and apple polyphenols. J. Agric. Food Chem..

[38] Anderson J.E., Beelman R.R., Doores S. (1996). Persistence of Serological and Biological Activities of Staphylococcal Enterotoxin A in Canned Mushrooms. J. Food Prot..

[39] Hu D.L., Nakane A. (2014). Mechanisms of staphylococcal enterotoxin-induced emesis. Eur. J. Pharmacol..

[40] Denny C.B., Tan P.L., Bohrer C.W. (1966). Heat Inactivation of Staphylococcal enterotoxin A. J. Food Sci..

[41] Bennett R.W. (1992). The biomolecular temperament of staphyloccal-enterotoxin in thermally processed foods. J. AOAC Int..

[42] Jamlang E.M., Bartlett M.L., Snyder H.E. (1971). Effect of pH, protein concentration, and ionic strength on heat inactivation of staphylococcal enterotoxin B 1. Appl. Microbiol..

[43] Humber J.Y., Denny C.B., Bohrer C.W. (1975). Influence of pH on the heat inactivation of staphylococcal enterotoxin A as determined by monkey feeding and serological assay. Appl. Microbiol..

[44] Tatini S.R. (1976). Thermal-Stability of Enterotoxins in Food. J. Milk Food Technol..

[45] Akhtar M., Park C.E., Rayman K. (1996). Effect of urea treatment on recovery of staphylococcal enterotoxin A from heat-processed foods. Appl. Environ. Microbiol..

[46] Reichert C.A., Fung D.Y.C. (1976). Thermal inactivation and subsequent reactivation of staphylococcal-enterotoxin b in selected liquid foods. J. Milk Food Technol..

[47] Read R.B., Bradshaw J.G., Dickerson R.W. (1965). Thermal inactivation of Staphylococcal enterotoxin B in raw milk. J. Dairy Sci..

[48] Read R.B., Bradshaw J.G. (1966). Staphylococcal enterotoxin B thermal inactivation in milk. J. Dairy Sci..

[49] Denny C.B., Humber J.Y., Bohrer C.W. (1971). Effect of toxin concentration on the heat inactivation of staphylococcal enterotoxin A in beef bouillon and in phosphate buffer. Appl. Microbiol..

[50] Tibana A., Rayman K., Akhtar M., Szabo R. (1987). Thermal-Stability of Staphylococcal Enterotoxins a, B and C in a Buffered System. J. Food Prot..

[51] Murzin A.G. (1993). OB(oligonucleotide/oligosaccharide binding)-fold: Common structural and functional solution for non-homologous sequences. EMBO J..

[52] Burroughs A.M., Balaji S., Iyer L.M., Aravind L. (2007). Small but versatile: The extraordinary functional and structural diversity of the beta-grasp fold. Biol. Direct..

[53] Spaulding A.R., Salgado-Pabon W., Kohler P.L., Horswill A.R., Leung D.Y., Schlievert P.M. (2013). Staphylococcal and streptococcal superantigen exotoxins. Clin. Microbiol. Rev..

[54] McElhaney R.N. (1982). The use of differential scanning calorimetry and differential thermal analysis in studies of model and biological membranes. Chem. Phys. Lipids.

[55] Losada-Perez P., Mertens N., de Medio-Vasconcelos B., Slenders E., Leys J., Peeters M., van Grinsven B., Gruber J., Glorieux C., Pfeiffer H. (2015). Phase Transitions of Binary Lipid Mixtures: A Combined Study by Adiabatic Scanning Calorimetry and Quartz Crystal Microbalance with Dissipation Monitoring. Adv. Condens. Matter Phys..

[56] Medved I., Jurci M., Trnik A. (2022). Determination of phase change temperature of materials from adiabatic scanning calorimetry data. J. Therm. Anal. Calorim..

[57] Roberts D., Keeling R., Tracka M., van der Walle C.F., Uddin S., Warwicker J., Curtis R. (2015). Specific ion and buffer effects on protein-protein interactions of a monoclonal antibody. Mol. Pharm..

[58] Collins K.D. (1997). Charge density-dependent strength of hydration and biological structure. Biophys. J..

[59] Ponikova S., Antosova A., Demjen E., Sedlakova D., Marek J., Varhac R., Gazova Z., Sedlak E. (2015). Lysozyme stability and amyloid fibrillization dependence on Hofmeister anions in acidic pH. J. Biol. Inorg. Chem..

[60] Stavropoulos P., Thanassoulas A., Nounesis G. (2018). The effect of cations on reversibility and thermodynamic stability during thermal denaturation of lysozyme. J. Chem. Thermodyn..

[61] Schad E.M., Zaitseva I., Zaitsev V.N., Dohlsten M., Kalland T., Schlievert P.M., Ohlendorf D.H., Svensson L.A. (1995). Crystal structure of the superantigen staphylococcal enterotoxin type A. EMBO J..

[62] Sundstrom M., Hallen D., Svensson A., Schad E., Dohlsten M., Abrahmsen L. (1996). The Co-crystal structure of staphylococcal enterotoxin type A with Zn^2+^ at 2.7 A resolution. Implications for major histocompatibility complex class II binding. J. Biol. Chem..

[63] Hakansson M., Petersson K., Nilsson H., Forsberg G., Bjork P., Antonsson P., Svensson L.A. (2000). The crystal structure of staphylococcal enterotoxin H: Implications for binding properties to MHC class II and TcR molecules. J. Mol. Biol..

[64] Petersson K., Hakansson M., Nilsson H., Forsberg G., Svensson L.A., Liljas A., Walse B. (2001). Crystal structure of a superantigen bound to MHC class II displays zinc and peptide dependence. EMBO J..

[65] Cavallin A., Arozenius H., Kristensson K., Antonsson P., Otzen D.E., Bjork P., Forsberg G. (2000). The spectral and thermodynamic properties of staphylococcal enterotoxin A, E, and variants suggest that structural modifications are important to control their function. J. Biol. Chem..

[66] Regenthal P., Hansen J.S., Andre I., Lindkvist-Petersson K. (2017). Thermal stability and structural changes in bacterial toxins responsible for food poisoning. PLoS ONE.

[67] Meechan P.J., Potts J. (2020). Biosafety in Microbiological and Biomedical Laboratories.

[68] US National Institutes of Health (NIH) (2019). NIH Guidelines for Research Involving Recombinant or Synthetic Nucleic Acid Molecules (NIH Guidelines).

[69] (2012). HHS Select Agents and Toxins. Revised. 42 CFR Part 73.

[70] Gao Y., Cao Z., Yang X., Abdelmegeed M.A., Sun J., Chen S., Beger R.D., Davis K., Salminen W.F., Song B.J. (2017). Proteomic analysis of acetaminophen-induced hepatotoxicity and identification of heme oxygenase 1 as a potential plasma biomarker of liver injury. Proteom. Clin. Appl..

[71] Goldberg R.N., Kishore N., Lennen R.M. (2002). Thermodynamic Quantities for the Ionization Reactions of Buffers. J. Phys. Chem. Ref. Data.

