# Supplementary material for: Microcalorimetric Investigations of Reversible Staphylococcal Enterotoxin Unfolding
Source: Toxins (Basel). 2022 Aug 15;14(8):554. doi: 10.3390/toxins14080554 (PMC9414061; doi:10.3390/toxins14080554)
Supplement: Supplementary file 1 [file toxins-14-00554-s001.zip › toxins-1838143-supplementary.pdf]

toxins

MDPI

Supplementary Materials: Microcalorimetric Investigations of Reversible Staphylococcal Enterotoxin Unfolding

Susan C. Berry, Odbert A. Triplett, Li-Rong Yu, Mark E. Hart, Lauren S. Jackson and William H. Tolleson *

Table S1. Deconvolution for SEA endothermic unfolding.

Buffer	Heating cycle	peak	ΔH_{cal} (kJ/mol)	ΔH_{vH} (kJ/mol)	$\frac{\Delta H_{cal}}{\Delta H_{vH}}$	T_m (ºC)
25 mM sodium acetate, pH 4.50	1	Total	419 ± 15	370 ± 2	1.1 ± 0.1	59.9 ± 0.2
		A	475 ± 156	244 ± 156	2.3 ± 1.6	58.9 ± 2.2
		B	575 ± 155	183 ± 155	3.6 ± 2	60.2 ± 2.6
	2	Total	55 ± 18	460 ± 80	0.1 ± 0.1	60.4 ± 1.9
		A	498 ± 197	32 ± 197	40 ± 50	67.5 ± 9.6
		B	646 ± 304	21 ± 304	60 ± 50	53 ± 11
25 mM sodium acetate, 10 µM zinc chloride, pH 4.50	1	Total	400 ± 20	340 ± 10	1.2 ± 0.1	60.7 ± 0.2
		A	391 ± 16	269 ± 16	1.5 ± 0.1	58.1 ± 0.2
		B	649 ± 28	136 ± 28	4.8 ± 0.5	62.3 ± 0.1
	2	Total	85 ± 6	330 ± 11	0.3 ± 0.1	61.5 ± 0.8
		A	304 ± 257	24 ± 257	45 ± 42	58.8 ± 1.4
		B	419 ± 118	64 ± 118	8.3 ± 6.5	62.3 ± 1.4
25 mM sodium phosphate, pH 6.80	1	Total	520 ± 13	390 ± 20	1.3 ± 0.1	65.8 ± 0.8
		A	417 ± 17	350 ± 17	1.2 ± 0.1	63.4 ± 0.3
		B	726 ± 23	172 ± 23	4.2 ± 0.4	67.3 ± 0.2
	2	Total	n.d.	n.d.	n.d.	n.d.
		A	n.d.	n.d.	n.d.	n.d.
		B	n.d.	n.d.	n.d.	n.d.
25 mM sodium phosphate, 10 µM zinc chloride, pH 6.80	1	Total	590 ± 40	460 ± 90	1.3 ± 0.2	66.3 ± 0.1
		A	478 ± 11	351 ± 11	1.4 ± 0.1	67.5 ± 0.1
		B	786 ± 24	221 ± 24	3.6 ± 0.3	71.6 ± 0.1
	2	Total	n.d.	n.d.	n.d.	n.d.
		A	n.d.	n.d.	n.d.	n.d.
		B	n.d.	n.d.	n.d.	n.d.

Table S2. Deconvolution for SEB endothermic unfolding.

Buffer	Heating cycle	peak	ΔH_{cal} (kJ/mol)	ΔH_{vH} (kJ/mol)	$\frac{\Delta H_{cal}}{\Delta H_{vH}}$	T_m (ºC)
25 mM sodium acetate, pH 4.50	1	Total	690 ± 60	410 ± 10	1.7 ± 0.2	73.5 ± 0.5
		A	431 ± 9	440 ± 9	1 ± 0.1	70.4 ± 0.3
		B	742 ± 40	268 ± 40	2.8 ± 0.5	74.5 ± 0.2
	2	Total	390 ± 10	370 ± 10	1.0 ± 0.1	71.8 ± 0.7
		A	509 ± 171	149 ± 171	6.6 ± 7.9	67.6 ± 1.9
		B	528 ± 80	237 ± 80	2.5 ± 1.1	72.5 ± 1.1
83 mM sodium acetate, pH 4.50	1	Total	650 ± 11	460 ± 10	1.4 ± 0.2	73.4 ± 0.4
		A	464 ± 1	415 ± 1	1.1 ± 0.1	70.2 ± 0.1
		B	870 ± 21	249 ± 21	3.5 ± 0.2	74.1 ± 0.1
	2	Total	122 ± 8	390 ± 10	0.3 ± 0.1	71.5 ± 0.4
		A	431 ± 23	93 ± 23	4.6 ± 0.2	69.9 ± 0.6
		B	835 ± 157	31 ± 157	31 ± 16	74 ± 0.4
25 mM sodium citrate, pH 4.50	1	Total	420 ± 5	500 ± 30	0.8 ± 0.1	72.0 ± 0.1
		A	459 ± 39	232 ± 39	2.0 ± 0.1	68.5 ± 0.2
		B	882 ± 58	185 ± 58	4.8 ± 0.5	72.3 ± 0.1
	2	Total	n.d.	n.d.	n.d.	n.d.
		A	n.d.	n.d.	n.d.	n.d.
		B	n.d.	n.d.	n.d.	n.d.
25 mM sodium acetate, 25 mM sodium phosphate, pH 4.50	1	Total	560 ± 40	530 ± 90	1.1 ± 0.1	73.6 ± 0.6
		A	491 ± 5	349 ± 5	1.4 ± 0.1	70.4 ± 0.1
		B	916 ± 9	194 ± 9	4.7 ± 0.1	74.2 ± 0.1
	2	Total	90 ± 10	340 ± 80	0.3 ± 0.1	71.2 ± 0.6
		A	486 ± 135	40 ± 135	14 ± 8	68.7 ± 0.1
		B	564 ± 34	46 ± 34	12.2 ± .6	73.3 ± .1
25 mM sodium acetate, 25 mM sodium phosphate, 50 mM imidazole, pH 4.50	1	Total	540 ± 80	490 ± 10	1.1 ± 0.2	73.5 ± 0.1
		A	398 ± 138	260 ± 138	4.0 ± 4.8	69.4 ± 1.6
		B	823 ± 90	166 ± 90	24 ± 35	73.8 ± 0.8
	2	Total	43 ± 8	350 ± 60	0.1 ± 0.1	71.6 ± 1.5
		A	462 ± 29	222 ± 29	4.6 ± 5.3	69.7 ± 0.1
		B	913 ± 43	138 ± 43	37 ± 55	73.8 ± 0.5
25 mM sodium phosphate, pH 6.80	1	Total	570 ± 70	430 ± 10	1.3 ± 0.2	70.8 ± 0.2
		A	478 ± 11	351 ± 11	1.4 ± 0.1	67.5 ± 0.1
		B	786 ± 24	221 ± 24	3.6 ± 0.3	71.6 ± 0.1
	2	Total	n.d.	n.d.	n.d.	n.d.
		A	n.d.	n.d.	n.d.	n.d.
		B	n.d.	n.d.	n.d.	n.d.
25 mM sodium citrate, pH 6.80	1	Total	510 ± 60	420 ± 30	1.2 ± 0.2	69.3 ± 0.7
		A	443 ± 49	325 ± 49	1.4 ± 0.4	66.4 ± 0.1
		B	768 ± 67	192 ± 67	4.1 ± 0.8	70.4 ± 0.1
	2	Total	n.d.	n.d.	n.d.	n.d.
		A	n.d.	n.d.	n.d.	n.d.
		B	n.d.	n.d.	n.d.	n.d.

Table S3. Deconvolution for SEB pH study endothermic unfolding.

Buffer	Heating cycle	peak	ΔH_{cal} (kJ/mol)	ΔH_{vH} (kJ/mol)	$\frac{\Delta H_{cal}}{\Delta H_{vH}}$	T_m (ºC)
25 mM sodium acetate, pH 3.50	1	Total	850 ± 70	530 ± 30	1.6 ± 0.2	71.1 ± 0.2
		A	479 ± 46	452 ± 46	1.1 ± 0.1	68.1 ± 0.5
		B	897 ± 49	392 ± 49	2.3 ± 0.4	71.5 ± 0.2
	2	Total	500 ± 50	330 ± 30	1.5 ± 0.2	66.3 ± .3
		A	338 ± 15	479 ± 15	0.7 ± 0.1	66.2 ± 0.1
		B	1343 ± 161	30 ± 161	47 ± 12	72.3 ± 0.1
25 mM sodium acetate, pH 3.75	1	Total	660 ± 80	500 ± 20	1.3 ± 0.1	72.7 ± 0.3
		A	462 ± 13	380 ± 13	1.2 ± 0.1	69.4 ± 0.1
		B	877 ± 19	283 ± 19	3.1 ± 0.4	73 ± 0.2
	2	Total	430 ± 40	360 ± 10	1.2 ± 0.1	68.6 ± 0.2
		A	371 ± 27	237 ± 27	2.3 ± 1.6	65.7 ± 2.0
		B	687 ± 442	204 ± 442	13 ± 20	71.0 ± 1.8
25 mM sodium acetate, pH 4.00	1	Total	660 ± 270	460 ± 20	1.4 ± 0.6	73.3 ± 0.5
		A	449 ± 40	289 ± 40	1.6 ± 0.2	70.3 ± 0.3
		B	844 ± 50	180 ± 50	4.7 ± 0.6	74.1 ± 0.2
	2	Total	460 ± 140	360 ± 10	1.3 ± 0.3	70.5 ± 0.3
		A	356 ± 19	187 ± 19	1.9 ± 0.1	67.8 ± 0.3
		B	480 ± 29	179 ± 29	2.7 ± 0.6	72.3 ± 0.2
25 mM sodium acetate, pH 4.25	1	Total	910 ± 140	450 ± 10	2.0 ± 0.3	73.7 ± 0.1
		A	455 ± 7	580 ± 7	0.8 ± 0.1	70.5 ± 0.1
		B	848 ± 17	344 ± 17	2.5 ± 0.5	74.4 ± 0.1
	2	Total	580 ± 130	380 ± 10	1.5 ± 0.3	71.1 ± 0.5
		A	394 ± 9	298 ± 9	1.4 ± 0.3	68 ± 0.3
		B	550 ± 5	291 ± 5	2.0 ± 0.6	72.6 ± 0.2
25 mM sodium acetate, pH 4.50	1	Total	690 ± 60	410 ± 10	1.7 ± 0.2	73.5 ± 0.5
		A	431 ± 9	440 ± 9	1 ± 0.1	70.4 ± 0.3
		B	742 ± 40	268 ± 40	2.8 ± 0.5	74.5 ± 0.2
	2	Total	390 ± 10	370 ± 10	1.0 ± 0.1	71.8 ± 0.7
		A	509 ± 171	149 ± 171	6.6 ± 7.9	67.6 ± 1.9
		B	528 ± 80	237 ± 80	2.5 ± 1.1	72.5 ± 1.1
25 mM sodium acetate, pH 4.75	1	Total	600 ± 100	440 ± 10	1.4 ± 0.2	74.0 ± 0.8
		A	481 ± 6	394 ± 6	1.2 ± 0.2	71 ± 0.3
		B	820 ± 62	214 ± 62	4.0 ± 1.3	74.9 ± 0.2
	2	Total	280 ± 70	400 ± 20	0.7 ± 0.2	72.5 ± 0.6
		A	443 ± 32	187 ± 32	2.5 ± 0.6	70.1 ± 0.5
		B	724 ± 57	92 ± 57	8.3 ± 2.5	74.2 ± 0.2
25 mM sodium acetate, pH 5.00	1	Total	490 ± 90	450 ± 20	1.1 ± 0.2	73.5 ± 0.2
		A	497 ± 7	346 ± 7	1.5 ± 0.4	71.2 ± 0.1
		B	873 ± 19	159 ± 19	5.6 ± 0.8	75 ± 0.1
	2	Total	70 ±30	410 ± 50	0.2 ± 0.1	72.9 ± 0.7
		A	583 ± 162	51 ± 162	26 ± 27	69.8 ± 2.5
		B	603 ± 62	34 ± 62	21 ± 12	74.5 ± 2.3
25 mM sodium acetate, pH 5.50	1	Total	540 ± 40	420 ± 20	1.3 ± 0.2	72.5 ± 0.8
		A	494 ± 48	365 ± 48	1.4 ± 0.5	70.6 ± 0.4
		B	814 ± 81	170 ± 81	5.0 ± 1.5	74.4 ± 0.4
	2	Total	n.d.	n.d.	n.d.	n.d.
		A	n.d.	n.d.	n.d.	n.d.
		B	n.d.	n.d.	n.d.	n.d.

Table S4. Deconvolution for SEH endothermic unfolding.

Buffer	Heating cycle	peak	ΔH_{cal} (kJ/mol)	ΔH_{vH} (kJ/mol)	$\frac{\Delta H_{cal}}{\Delta H_{vH}}$	T_m (ºC)
25 mM sodium acetate, pH 4.50	1	Total	310 ± 80	430 ± 80	0.7 ± 0.2	73.8 ± 0.2
		A	415 ± 38	194 ± 38	2.2 ± 0.7	69.5 ± 0.5
		B	912 ± 33	126 ± 33	7.4 ± 1.5	73.9 ± 0.1
	2	Total	n.d.	n.d.	n.d.	n.d.
		A	n.d.	n.d.	n.d.	n.d.
		B	n.d.	n.d.	n.d.	n.d.
25 mM sodium acetate, 10 µM zinc chloride, pH 4.50	1	Total	400 ± 40	490 ± 30	0.8 ± 0.1	73.6 ± 0.5
		A	411 ± 65	254 ± 65	1.6 ± 0.1	70.3 ± 0.8
		B	934 ± 19	157 ± 19	6.6 ± 2.4	73.8 ± 0.3
	2	Total	n.d.	n.d.	n.d.	n.d.
		A	n.d.	n.d.	n.d.	n.d.
		B	n.d.	n.d.	n.d.	n.d.
25 mM sodium phosphate, pH 6.80	1	Total	280 ± 20	400 ± 20	0.7 ± 0.1	62.1 ± 0.3
		A	390 ± 22	174 ± 22	2.3 ± 0.4	57.6 ± 0.2
		B	784 ± 16	116 ± 16	6.8 ± .1	62.3 ± 0.1
	2	Total	160 ± 20	350 ± 50	0.5 ± 0.1	62.2 ± 0.4
		A	333 ± 24	113 ± 24	3.0 ± 0.6	56.3 ± 0.2
		B	737 ± 2	57 ± 2	13 ± 1.3	62.2 ± 0.1
25 mM sodium phosphate, 10 µM zinc chloride, pH 6.80	1	Total	520 ± 40	480 ± 80	1.1 ± 0.1	61.9 ± 0.1
		A	404 ± 15	303 ± 15	1.3 ± 0.1	57.6 ± 0.5
		B	789 ± 20	231 ± 20	3.4 ± 0.4	61.9 ± 0.2
	2	Total	370 ± 20	290 ± 10	1.1 ± 0.1	60.6 ± 1.3
		A	287 ± 21	237 ± 21	1.2 ± 0.1	55 ± 0.8
		B	640 ± 42	137 ± 42	4.7 ± .5	61.4 ± 0.5
